# Exploring motivations and resistances for implementing shared decision‐making in clinical practice: A systematic review based on a structure–process–outcome model

**DOI:** 10.1111/hex.13541

**Published:** 2022-06-05

**Authors:** Changhai Tang, Anqi Wang, Jingjing Yan

**Affiliations:** ^1^ School of Public Health Weifang Medical University Weifang Shandong China; ^2^ College of Philosophy Law & Political Science, Shanghai Normal University Shanghai China; ^3^ School of International and Public Affairs Shanghai Jiao Tong University Shanghai China

**Keywords:** motivation, patient‐centred care, resistance, shared decision‐making, structure–process–outcome model

## Abstract

**Objective:**

Shared decision‐making (SDM) as a multicollaborative approach is vital for facilitating patient‐centred care. Considering the limited clinical practice, we attempted to synthesize the motivations and resistances, and investigate their mutual relationships for advancing the implementation of SDM.

**Methods:**

A comprehensive systematic review using Preferred Reporting Items for Systematic Review and Meta‐Analysis guidelines was performed. ‘Shared decision making’ was searched as the mesh term through PubMed, Web of Science and EBSCO from 2000 to 2021, and the quality of literature was appraised using the QualSyst Tool. Motivations and resistances were categorized based on content analysis and the ‘structure–process–outcome’ model.

**Results:**

From 8319 potential citations, 105 were included, comprising 53 qualitative studies (the average quality score is 0.92) and 52 quantitative studies (the average quality score is 0.95). A total of 42 categories of factors were identified into 11 themes and further grouped into three dimensions: structure, process and outcome. The structure dimension comprised six themes (71.43%), the process dimension contained four themes (11.01%) and the outcome dimension covered only one theme. Across all categories, decision‐making time and patients' decision preparedness in the process dimension were the most reported, followed by physicians' communication skills and health care environment in the structure dimension. Analysis of implementation of SDM among various types of diseases showed that more influencing factors were extracted from chronic diseases and unspecified disease decisions.

**Conclusions:**

The major determinants for the implementation of SDM are focused on the structural dimension, which challenges the health systems of both developed and low‐ and middle‐income countries. Furthermore, we consider it important to understand more about the interactions among the factors to take integrated measures to address the problems and to ensure the effectiveness of implementing SDM.

**Patient or Public Contribution:**

Patients, healthcare professionals and other stakeholders articulated their perspectives on the implementation of SDM actively, and these were adopted and analysed in this study. However, the above‐mentioned individuals were not directly involved in the process of this study. Protocol was registered on PROSPERO (CRD42021259309).

## 1 INTRODUCTION

Shared decision‐making (SDM) has gained traction in the patient‐centred care model and is recognized as an advanced and multicollaborative approach to decision‐making practice by combining the clinical evidence and patient preferences in mature healthcare systems.^1^, ^2^ Charles et al.^3^ stated that SDM involved at least two participants, the physician and the patient, and further elaborated that SDM included the following characteristic elements: starting with the physicians' medical diagnosis for the patient, followed by a thorough discussion between the physician and the patient, including clarification of each treatment option, analysis of benefits and harms, and patient preferences, and then ending with a physician–patient shared treatment decision. Although the latest studies have incorporated the diagnoses and opinions of nurses and pharmacists into the process of SDM, the view of Charles et al.^3^ is still well accepted and practiced.^4^ We must emphasize that physicians' medical diagnosis and treatment decisions are an indispensable part of SDM. Moreover, nurses' diagnosis can complement the medical process as an information gatherer, coach, advisor, negotiator or caretaker. We consider medical diagnosis and decision as an important foundation of the study.

SDM advocates physicians and patients seeking consensus on treatment options via immersive participation.^5^ By providing accessible initiatives for information sharing and exchanging, physicians explain treatment options and therapeutic risks, and patients elucidate their symptoms, treatment preferences and health expectations at the same time.^6^, ^7^ Enabling more voices from patients, SDM stresses the importance of physician–patient communication in health performance and economic benefits.^8^, ^9^ It also poses challenges for physicians in encouraging patients' participation in decisions, addressing the differences between patient values and clinical practice guidelines and chasing personalized and tailored treatment plans depending on patients' characteristics.^10^

SDM has been gaining prominence with increasing patient self‐awareness, and increasing access to evidence‐based knowledge regarding diagnosis, complex treatment options, risk communication and value assessment, which occurred as a prerequisite for patients to play an active role and share in the responsibility with the physicians in medical decisions.^11^ SDM has been officially integrated into clinical practice in several countries. Health authorities have enacted the *Patient Protection and Affordable Care Act* in the United States,^12^ included patient representatives in the *National Act* in the United Kingdom,^13^ and included SDM in its social health insurance programmes in Germany.^14^ Even though a growing number of authorities and medical professionals were adopting SDM as their preferred approach, it was still minimally used in clinical practice. It was estimated that only 10% of health decisions were made using the SDM model.^15^ A report by the Institute of Medicine stated that physicians sought patients' treatment preferences probably half the time of the diagnostic procedure.^11^ Implementation of SDM into daily practice still has far to go to fulfil its real promise.

Understanding the motivations and resistances, such as time constraints, communication difficulties, mutual trust and adequate training,^16^ is paramount in the implementation of SDM. Thus, we logically reason that for effective healthcare, an overview of the factors affecting its implementation should be considered. The literature  on the factors of motivation and resistance to SDM is vast, and the taxonomy of factors was developed by Joseph‐Williams et al.,^17^ and coded into the following categories: predisposing factors, interactional context factors, preparation for SDM encounters or processes and its stakeholders, while few measured elements including decision antecedents, decision processes and decision outcomes were taken into account for categories. Moreover, a couple of studies focused on specific practices in oncology,^11^ mental health^18^ or targeted groups of the elderly^15^ or the youth,^19^ lacking integrated views on clinical practice. Therefore, we included these in our methodological coding.

To gain more insight into the factors that motivate and resist the implementation of SDM, we conducted a comprehensive systematic review to categorize existing information with factors influencing SDM, based on the ‘structure–process–outcome’ (SPO) model designed to evaluate the quality of healthcare in terms of compatibility with SDM measurement elements. With the consideration of these, this systematic review aims to (i) explore the main factors affecting the practice of SDM concerning the classification, (ii) explain the reasons for the effect of the main factors on SDM and the interaction mechanisms between these factors and (iii) aspire to guide the development in the future practice of SDM.

## 2 METHODS

### 2.1 Theoretical framework

Donabedian^20^ proposed the ‘SPO’ model to evaluate healthcare service quality focusing on context, actions and effects.^21^ As SDM is a participatory process related to distinguishing characteristics of ‘antecedents’, ‘processes’ and ‘outcomes’, the SPO model can be used as the categorization framework to identify factors influencing the implementation of SDM. The linear interaction inherent with the SPO model elucidates either the state of the structure, process and outcome of the research object or the logical relationship between the three.

### 2.2 Search

**Figure 1:**
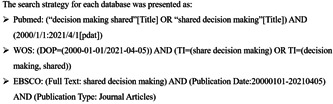
Search strategy.

This systematic review was registered on PROSPERO (CRD42021259309) and followed the Preferred Reporting Items for Systematic Review and Meta‐Analysis (PRISMA) guidelines (2009) (see File S1).^22^ Given that SDM was an important approach to healthcare in the late 1990s,^23^ relevant articles from 1 January, 2000 to 5 April 2021, were searched, evaluated and combed, databases like PubMed, Web of Science and EBSCO were searched systematically and ‘shared decision making’ as a Mesh term and broad derivatives such as ‘adaptive decision making’, ‘shared strategies’ and ‘shared countermeasures’ were used to ensure the integrity of the retrieved information. The full search strategy is shown in Figure 1.

### 2.3 Inclusion and exclusion criteria

Studies eligible for inclusion were (i) empirical studies using qualitative or quantitative investigation, (ii) results on motivations for and/or resistance to implementing SDM, (iii) conclusions concerning the relationship and/or interaction with patients and/or their families and healthcare professionals and (iv) manuscripts written in English.

Studies were excluded if (i) the manuscript was a literature review, (ii) motivations and/or resistance were not stated as an influencing factor in the implementation of SDM (e.g., preferences for implementation), (iii) studies considered SDM as a physician‐only or patient‐only process or (iv) manuscripts were not in English.

### 2.4 Study selection

We excluded duplicated articles after exporting to endnote software. Then, we allocated articles to two reviewers, who conducted the first screening of titles and abstracts with the study objectives, and judged whether an article was ‘included’, ‘excluded’ or ‘inconclusive’ depending on the criteria. If two reviewers made inconsistent judgements for the same article, the third reviewer would conduct a second screening and give the final answer.

### 2.5 Data collection process and data items

We implemented two measures to extract information accurately. First, reviewers were trained before the data collection to reach uniform norms on information extraction. Second, 10 articles were selected randomly for preanalysis first. Third, the remaining studies were coded independently according to the SPO framework in a back‐to‐back fashion by two reviewers with a standardized information extraction form, including background information (e.g., author, year), research information (e.g., designs, methods), participants (e.g., patients, physicians, stakeholders) and key findings (e.g., barriers and facilitators) (see File S2). Following the Cochrane Handbook of Systematic Reviews (2008),^24^ disagreements were resolved by a third reviewer's intervention to promote consensus during data extraction.

### 2.6 Study quality assessment

Two reviewers evaluated the quality of the included studies, following the quantitative and qualitative evaluation standards on the QualSyst tool,^25^ which is an accurate and preferred evaluation standard in the medical literature. If there was an inconsistency, a third reviewer would preside over the consultation and made the final decision. There were 14 evaluation items for quantitative studies and 10 evaluation items for qualitative studies in the QualSyst tool. Each item's score was as follows: ‘yes’ = 2, ‘partial’ = 1 and ‘no’ = 0, and items not suitable for the specified study design were labelled ‘N/A’ and eliminated from the calculation of the aggregated score. The aggregated score was calculated by totalling the relevant items and dividing the total possible scores (the total possible scores = 28 − [number of ‘N/A’ × 2]) for each quantitative study. Qualitative studies were scored similarly to quantitative studies (the total possible scores = 20 − [number of ‘N/A’ × 2]).

### 2.7 Data synthesis and presentation

As SDM was a ‘process‐oriented’ service, the motivations and resistances could be transformed in different situations, so we calculated the frequency depending on the situation in which the factor was positioned. If the article reported the motivation or the resistance repeatedly, we counted it only once. If the factor was distinguished as motivations and resistances for implementing SDM in different situations, we counted it for twice. If the factor was identified both as the motivation and as the resistance, we counted it for twice. If the study involved multiple subjects and the same factor was reported separately as a motivation or resistance for different subjects, it was counted separately for each subject.

## 3 RESULTS

### 3.1 Results of the search

**Figure 2:**
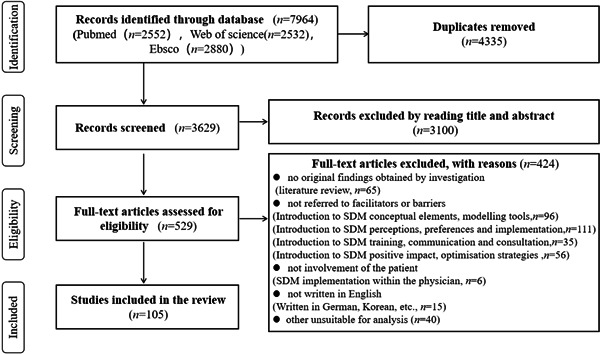
Preferred Reporting Items for Systematic Review and Meta‐Analysis flow diagram. SDM, shared decision‐making.

A total of 8319 articles were searched across the three electronic databases. After excluding 4690 duplicating articles, we further excluded 3100 articles that did not match our selection criteria. We screened the remaining 529 articles according to the mentioned inclusion and exclusion criteria, and a total of 105 articles were included (see Figure 2).

### 3.2 Study characteristics

A total of 53 studies (50.47%) were qualitative studies, and the rest of the 52 studies were quantitative studies. Response rates for the 105 studies ranged from 14.35% to 100%, of which 58 studies (55.24%) reported a response rate of over 75%. Most studies (*n* = 64, 60.95%) were published beyond 2015, but 31 studies (29.52%) were published from 2011 to 2015. Articles were from 17 countries, in which USA accounted for the most (*n* = 33, 31.43%), followed by Canada (*n* = 14, 13.33%), the Netherlands (*n* = 14, 13.33%) and the United Kingdom (*n* = 13, 12.38%). In terms of distribution by region, North America accounted for the largest proportion (*n* = 47, 44.76%), followed by Europe (*n* = 41, 39.05%). Factors of motivation and resistance were mostly reported in terms of the perspectives of patients (*n* = 32, 30.48%), followed by physicians (*n* = 18, 17.14%) and patient and physician (*n* = 18, 17.14%). 20.94% of studies focused on other subjects, covering professionals, stakeholders and nurses. Apart from seven studies (6.67%) that did not indicate any subject, the remaining studies synthesized 112,141 participants, including 103,026 patients, 3442 physicians, 1627 informants, 201 nurses and 935 additional types of healthcare professionals. In these studies, the proportion of females ranged from 16.67% to 100% (see Table 1).

**Table 1:** Characteristics of the included studies

Entry	Types	*N*	Percent (%)	Entry	Types	*N*	Percent (%)
Methods	Qualitative	53	50.48	Country	USA	33	31.43
	Quantitative	52	49.52		Canada	14	13.33
Year	2000–2005	3	2.86		The Netherlands	14	13.33
	2006–2010	8	7.62		UK	13	12.38
	2011–2015	31	29.52		Germany	9	8.57
	2016–2021	64	60.95		China	7	6.67
Female (%)	16.67–30	3	2.86		Other countries	15	14.29
	31–45	12	11.43	Region	North America	47	44.76
	46–60	27	25.71		Europe	41	39.05
	61–75	21	20.00		Asia	14	13.33
	76–90	11	10.48		Oceania	2	1.90
	91–100	7	6.67		Africa	1	0.95
	Not report	24	22.86	Subject	Patients	32	30.48
Response rate (%)	14.35–30	6	5.71		Physicians	18	17.14
	31–45	7	6.67		Patients and physicians	18	17.14
	46–60	7	6.67		Professionals	9	8.57
	61–75	13	12.38		Stakeholders	6	5.71
	76–90	19	18.10		Physician and nurse	5	4.76
	91–100	39	37.14		Nurse	2	1.90
	Not report	14	13.33		Not report	7	6.67
Factors	Barriers (B)	25	23.81		Others	8	7.62
	Facilitators (F)	27	25.71	Sample sizes	Patients	103,026	
	B & F	53	50.48		Physicians	3442	
Research background	Healthcare	59	56.19		Stakeholders	2517	
	Institution	27	25.71		Informants	1627	
	Clinical case	9	8.57		Professionals	935	
	Other	9	8.57		Family members	393	
	Not report	1	0.95		Nurses	201	

Most studies reported both factors of motivation and resistance in implementing SDM (*n* = 53, 50.47%), with 25 studies mentioning only the resistance (23.81%) and 27 studies mentioning only motivations (25.71%). Also, most studies based on healthcare related to patient topics (*n* = 59, 56.19%), followed by status surveys on institutions (*n* = 27, 25.71%) and nine studies (8.57%) based on clinical cases (see Table 1).

### 3.3 Quality assessment

The quality scores of the quantitative studies ranged from 0.75 to 1.00, and those of the qualitative studies ranged from 0.55 to 1.00 (see File S3).

In terms of the quantitative studies, inter‐rates of agreement ranged from 73.08% to 100% (see File S3). The quality scores assessed by the first reviewer ranged from 0.83 to 1.00 (mean = 0.96, standard deviation = 0.05), and for the second reviewer, these ranged from 0.75 to 1.00 (mean = 0.93, standard deviation = 0.05); the average score for quality assessment was 0.95. For 53 qualitative studies, the rates of agreement between the two reviewers ranged from 75.47% to 100% (see File S3). The quality score for the first reviewer ranged from 0.55 to 1.00 (mean = 0.94, standard deviation = 0.12), and that for the second one ranged from 0.55 to 1.00 (mean = 0.89, standard deviation = 0.12); the average score for quality assessment was 0.92.

### 3.4 Factors of motivation for and resistance to implementing SDM

**Figure 3:**
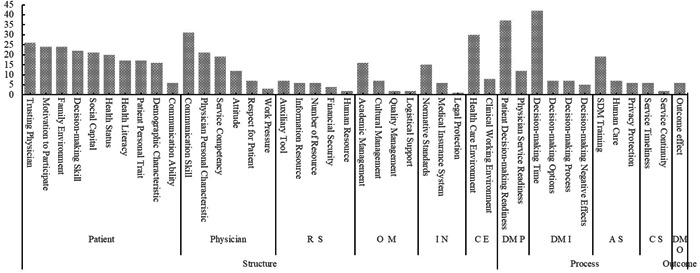
Histogram of SDM motivation and resistance. SDM, shared decision‐making.

**Figure 4:**
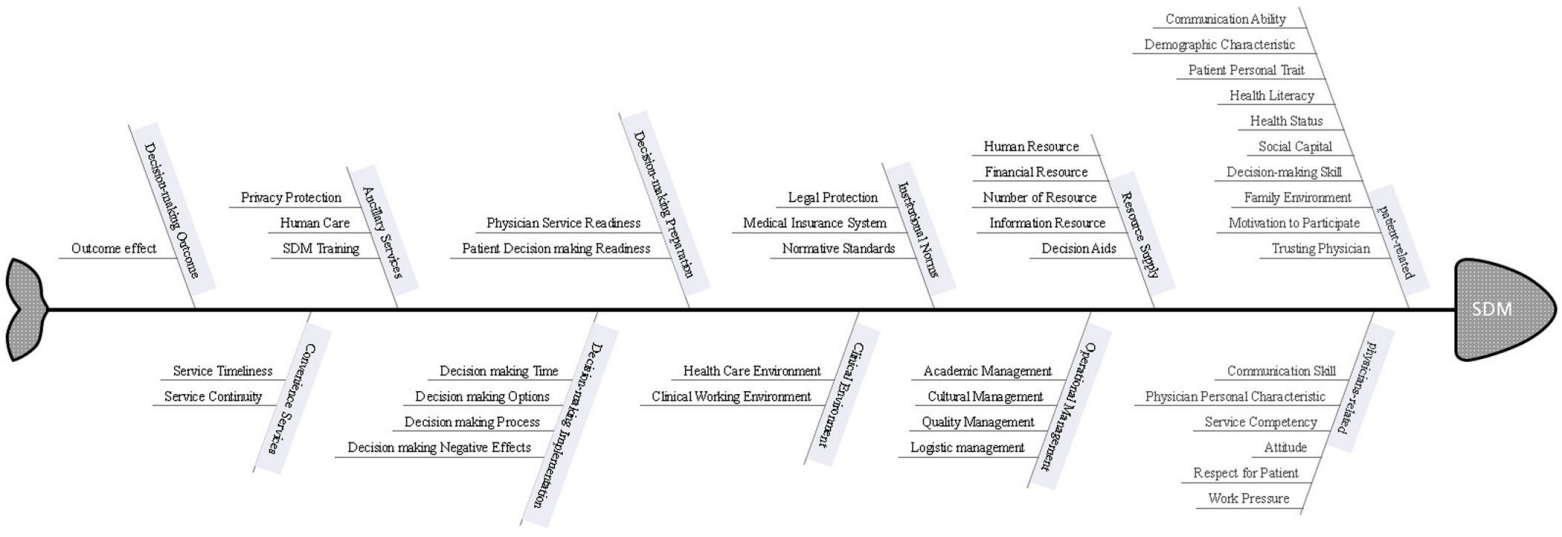
Fishbone diagram of SDM motivation and resistance. SDM, shared decision‐making.

From the 105 included studies (references in File S4), we extracted 269 factors that acted as motivators for and/or resistance to the implementation of SDM, with a total cumulative frequency of 554. According to the SPO model,^26^ the factors were grouped into 11 themes and 42 categories, which are presented in Table 2. Figures 3 and 4 show the distribution of factors.

**Table 2:** Categorization of motivation and resistance to SDM implementation

Level	Theme	Factor category	Including factors (examples）	Citations no.^a^	Frequency				
					Type 1	Type 2	Type 3	Type 4	Total
Structure	Patient‐related	Trusting physician	Confidence in physician, respecting physician, accrediting abilities of physician, mindlessly following physicians' authority…	24, 25, 29, 33, 34, 39, 40, 43, 49, 52, 55, 58, 59, 64, 67, 77, 82, 86, 88, 100, 112	3	5	10	8	26
		Motivation to participate	Positive attitudes, willingness, interest, adherence of patients to participate in SDM…	25, 26, 33, 40, 41, 44, 47, 51‐52, 55, 58, 59, 68, 71, 73, 88, 111, 112, 114, 116, 129	4	6	8	6	24
		Family environment	Level of support and emotional response of family members to SDM, family experience, level of family education…	24, 27, 29, 33, 38, 40, 52, 55–56, 58, 69, 71, 74, 82, 94, 102, 121, 122, 129, 137	2	7	8	7	24
		Decision‐making skill	Ability to obtain and receive information, ability to make decisions and choices…	25, 29, 43, 44, 47‐49, 55, 57, 59, 73, 80, 94, 100, 112, 115, 117, 129	4	5	8	5	22
		Social capital	Education level, employment status, socioeconomic status, interpersonal disease burden…	28, 30, 31, 35, 53, 54, 64, 70, 91–92, 110, 112, 113, 119, 122‐124, 130	1	3	9	8	21
		Health status	Experience of illness, state of health, length of illness…	28, 31, 34, 35, 52, 55, 70, 90, 94, 95, 102, 109, 115, 123, 129, 130	2	6	9	3	20
		Health literacy	Quality to access and understand healthy information, quality to process information for maintaining health…	30, 33, 34, 47, 56, 73, 79, 84, 86–88, 90, 91, 100, 112, 126, 129	6	2	8	1	17
		Personal trait	Personality, values, self‐efficacy, self‐awareness, acting style…	32‐34, 38, 40, 41, 46, 67, 69, 88, 113, 119, 129	3	3	10	1	17
		Demographic characteristic	Gender, age, race, education, language…	28, 31, 35‐37, 55, 64, 70, 79, 83, 86, 92, 110, 112, 129	3	2	6	5	16
		Communication ability	Expressive skills, language barriers…	33,47, 55, 83, 111, 129	2	1	3	0	6
	Physician‐related	Communication skill	Verbal skills for making professional information intelligible to patients, presentation skills for calming patients down, expression skills for inspiring patients to participate in SDM…	24, 34, 38, 40, 41, 43, 46, 47, 55–57, 61, 69, 71, 73, 75‐76, 80, 81, 88, 90, 99, 103, 105, 111, 121, 124, 127	2	5	14	10	31
		Personal characteristic	Leadership, acting style, values, mindset, self‐confidence…	32, 38, 43, 47, 49, 52, 56, 71, 77, 80, 89, 97, 111, 119, 124	2	0	10	9	21
		Service competency	Proficiency in the application of SDM tools, strong medical knowledge skills, good listening and judgement skills…	29, 33, 34, 46‐48, 54, 56, 66, 67, 80, 81, 93, 97, 99, 104, 130	4	2	10	3	19
		Attitude	Positive or negative attitudes towards SDM, supportive or sceptical attitudes towards SDM…	25, 33, 47, 49, 67, 77, 81, 101, 111, 122	2	0	7	3	12
		Respect for patient	Understanding of patient responsibility, respect for patients, the ability to place oneself in patients' shoes…	43, 47, 66, 96, 99, 117	0	3	2	2	7
		Work pressure	Higher workload…	42, 47, 118	0	0	1	2	3
	Resource supply	Decision aids	Availability of decision aids to provide medical information for patients, applicability of decision aids to assist in physician–patient communication…	25, 40, 44, 47, 51, 55, 131	1	2	2	2	7
		Information resources	SDM information system, elements, quantities…	40, 42, 43, 77, 80, 103	0	1	1	4	6
		Number of resources	The number of resources available…	41, 87, 99, 116, 122	2	1	3	0	6
		Financial resources	Financial support, income, loss…	43, 45, 68	2	0	0	2	4
		Human resources	Specialization of human resources, size of the workforce…	33, 46	0	0	2	0	2
	Operational management	Academic management	Management of disciplines' collaboration, interaction, exchange…	25, 33, 40, 42, 45, 47, 48, 51, 52, 55, 56, 58, 118, 121	4	3	4	4	15
		Cultural management	Management of patient‐centred culture, service innovation culture, teamwork culture, SDM support culture…	41, 45, 47, 48, 51, 57, 78	1	1	3	1	6
		Quality management	Management of care quality…	35, 75	0	0	0	1	1
		Logistic management	Management of environment, equipment…	47, 57	3	2	7	4	16
	Institutional norms	Normative standards	Availability of SDM explicit normative standards, guidelines, implementation frameworks…	25, 40, 48, 51, 55, 82, 87, 97, 101, 122	2	1	1	3	7
		Medical insurance system	Medical insurance coverage, percentage of support…	25, 28, 47, 48, 68, 122	1	1	0	0	2
		Legal protection	Availability of SDM legal…	49	1	0	1	0	2
	Therapeutic environments	Health care environment	Atmosphere of physician–patient relationship, environment of consultation services, language communication patterns…	25, 34, 41–43, 46, 50, 53, 56, 71, 80, 81, 89, 98, 102, 103, 115, 117, 120, 129, 131	3	4	11	12	30
		Clinical working environment	Nature of clinical work, internal and external conditions…	25, 46, 49, 51, 68, 80, 106	1	1	2	4	8
Process	Decision‐making preparation	Patient decision‐making readiness	Sound mental preparation, knowledge preparation, preparedness for the process, objectives, needs…	29, 34, 38, 40, 41, 43, 46, 47, 48, 52, 55, 57–59, 71, 73, 102, 109, 111–115, 117, 124, 129	8	7	11	11	37
		Physician service readiness	Knowledge of SDM processes, understanding of patient disease and medical needs…	34, 40, 47, 66, 68, 71, 76, 81, 98	1	3	6	2	12
	Decision‐making implementation	Decision‐making time	Taking too much time on SDM…	24, 25, 33, 36, 40, 41, 43, 44, 46, 47, 49, 51, 54, 55, 57, 59, 72, 73, 77, 80, 81, 87, 88, 90, 93, 97, 103, 106, 107, 111, 116, 117, 119, 122, 124, 128, 129	5	6	16	15	42
		Decision‐making options	Quantity of SDM options, difficult to choose between multiple options, cost of options…	47, 50, 54, 53, 82	0	0	4	3	7
		Decision‐making process	SDM process of complexity, uncertainty…	47, 53, 57, 71, 97, 108, 118	0	1	1	5	7
		Decision‐making negative effects	Not complying with clinical practice guidelines, causing patients to question or feel uncomfortable…	47, 55	0	1	4	0	5
	Ancillary services	SDM training	Availability of training programme, insufficient training hours, lack of training knowledge…	25, 40, 42, 43, 47‐49, 55, 56, 61, 67, 78, 80, 85, 101, 111, 124	3	2	9	5	19
		Human care	Giving emotional support to patients, considering patients' needs…	3, 57, 71, 98, 102, 120, 122	0	1	2	4	7
		Privacy protection	Having a separate private space to hold conversations…	33, 41, 43, 52, 80, 111	1	1	3	1	6
	Convenience services	Service timeliness	Early access to physicians when patient need, reducing patient waiting times…	25, 34, 40, 42, 43, 71	1	1	2	2	6
		Service continuity	Guidance service before treatment, effective service at the time of treatment, follow‐up service after treatment…	103, 124	0	0	1	1	2
Outcome	Decision‐making outcome (DMO)	Outcome effect	A benefit cycle contributing to the next SDM…	47	0	1	5	0	6

Abbreviation: SDM, shared decision‐making.

^a^ The citations no. corresponded to the File S4. References for all. DOC

#### 3.4.1 Structure level‐Theme 1: Patient‐related

Ten categories were summarized under the patient‐related theme, with the top five categories being ‘trusting physicians’, ‘motivation to participate’, ‘family environment’, ‘decision‐making skill’ and ‘social capital’.

‘Trusting physicians’ indicates a greater possibility for patients to participate in decision‐making within trusting relationships,^27^ while it appears to be negative when patients trust physicians unconditionally without a reciprocal relationship,^28^ besides improving the motivation of participation facilitates for implementing SDM, and it is associated with the degree of family support and patients' social capital.^29^, ^30^, ^31^ Additionally, patients with better physical health, decision‐making skills and high health literacy are more easily involved in the adoption of SDM.^32^, ^33^, ^34^ Patients with open, honest, polite, confident personalities, higher self‐efficacy and self‐awareness are more likely to make decisions.^35^, ^36^, ^37^ Also, patients who are younger, less susceptible to racial prejudice and have few language barriers are considered more likely to participate in SDM.^38^, ^39^, ^40^

#### 3.4.2 Structure level‐Theme 2: Physician‐related

One of the most powerful motivators for the use of SDM is physicians' communication skills, which is characterized as a more important contributing factor at a ratio of 2:1 than their service competency.^37^, ^41^ Also, patients prefer female and white physicians to provide medical decisions.^42^ Physicians who are open, calm and empathetic make patients feel more comfortable, and this may enable patients to feel respected and they may be honest in sharing views.^43^, ^44^ Physicians with a positive attitude to SDM, and less work pressure are more likely to implement SDM.^28^, ^45^, ^46^

#### 3.4.3 Structure level‐Theme 3: Resource supply

Significant gains in information by using Decision Aids can promote decision engagement and improve treatment adherence compared to routine care,^47^ while limited available resources, such as information resources, financial resources and human resources, are seen as critical factors in bridging the divide between the practical and ideal performance of SDM.^45^, ^48^, ^49^

#### 3.4.4 Structure level‐Theme 4: Operational management

Operational management could help to pursue the preferred treatment option in the process of SDM,^36^, ^38^, ^50^, ^51^ which corresponds with the management of multidisciplinary dialogue, professional and competitive culture, high‐quality care and logistics support.

#### 3.4.5 Structure level‐Theme 5: Institutional norms

Kanzaria et al.^52^ and McCarter et al.^51^ reported that the absence of law or normative standards covering guidelines and implementation frameworks would prevent SDM from achieving the desired results. In addition, the execution of SDM may not be successful in situations where patients have limited health insurance coverage.^31^

#### 3.4.6 Structure level‐Theme 6: Therapeutic environments

The mutual respect and equitable treatment between patients and physicians are necessary elements that constitute a harmonious clinical environment, and essential requirements for the construction of a patient‐centred service model, which will admittedly enhance the effectiveness of the practice of SDM.^53^, ^54^

#### 3.4.7 Process level‐Theme 1: Decision‐making preparation

Patients who are adequately informed and prepared for their conditions have the confidence to take responsibility for their treatment decisions more easily and are thus inclined to be involved in SDM.^55^ Similarly, physicians with full knowledge of SDM processes, and motivated to understand the patients' diseases and medical needs are more open to engaging in the use of SDM.^37^

#### 3.4.8 Process level‐Theme 2: Decision‐making implementation

Physicians devote enormous time to consulting and discussing the process of SDM, including providing patients with a variety of possible treatment options, and explaining the uncertainties and complexities of each option that require to be overcome. It requires a significant amount of time for the physician to implement SDM effectively, while the time invested by physicians in SDM is only the ‘tip of the iceberg’.^4^, ^43^, ^56^, ^57^

#### 3.4.9 Process level‐Theme 3: Ancillary services

The absence of SDM training could serve to compromise the authenticity of SDM.^58^ Similarly, lacking humanistic concern can lead to the possibility for ineffective SDM, which should not be discounted.^59^ Moreover, given the lack of a separate space for privacy when communicating with physicians, patients do not provide detailed information on their condition, which is a major impediment to SDM.^44^

#### 3.4.10 Process level‐Theme 4: Convenience services

The lack of timeliness and continuity in services is a possible aspect being overlooked and consequently not easily appreciated. Being concerned with continuous and timely services can be regarded as an advantage of SDM.^28^, ^37^

#### 3.4.11 Outcome level‐Theme 1: Decision‐making outcome

SDM helps to provide better options for both physicians and patients, and contributes to the improvement of patient compliance, ultimately leading to a better physician–patient relationship, in turn promoting further practice of SDM.^50^

### 3.5 Factors' distribution among different types of articles

Sorting through the included literature, it was found that the process of SDM was mainly used in four categories of diseases (see Table 2).

The first type, with 19 articles, dealt with SDM in the cancer treatment process. The factors reported in these articles covered 31 categories, with a cumulative frequency of factors reaching 80, including 30 times for factors under the theme of patient‐related and 10 times for the physician‐related theme. Patients' decision‐making readiness, health literacy and decision‐making time are the top three factors screened for these types of articles.

The second type consisted of 16 articles, which reported on factors influencing SDM for mental health disorders. The screened factors covered 31 categories with a cumulative frequency of 91 times, including 40 times for factors under the theme of patient‐related and 10 times for the physician‐related theme. Factors of patients' family environment, patients' decision‐making readiness, decision‐making time, patients' motivation to participate and patients' health status had reached the frequencies five times or more.

The third type, with 32 articles, revolved around factors influencing SDM in the treatment of chronic diseases (other than cancer and mental health disorders), such as hypertension, diabetes and other diseases. Among these studies, factors influencing the implementation of SDM included 39 categories with a cumulative frequency of 224 times, and factors in the patient‐related theme 79 times, physicians‐related theme 44 times and decision‐making implementation 25 times. Eight categories of factors, including decision‐making time, physicians' communication skills, health care environment, patients' decision‐making readiness, patients' trust in physicians, patients' personal traits, physicians' personal characteristics and physicians' service competency, all reached a cumulative frequency of 10 times or more.

In the fourth type, with 38 articles, the focus was on the factors influencing SDM in treatment for certain unspecified diseases. The screened factors covered 35 categories with cumulative frequencies of 159 times, in which the cumulative frequencies under the patient‐related theme reached 44 times, physician‐related theme reached 29 times and decision‐making implementation reached 23 times. Four categories of factors, including decision‐making time, health care environment, patients' decision‐making readiness and physicians' communication skills, all had frequencies of 10 times or more.

Furthermore, as the distribution among different types of studies for the same elements showed, the third and fourth types of studies were predominant.

## 4 DISCUSSION

We identified 105 studies, comprising 53 qualitative studies and 52 quantitative studies, reporting motivation and resistance based on the SPO model to promote the process of SDM.

Patients trusting physicians were the most frequently mentioned factor category under the patient‐related theme in the structure dimension; research showed that adequate trust could greatly improve the physician–patient relationship and make patients feel more comfortable in sharing their preferences, values and priorities within the trusting relationships,^27^, ^60^, ^61^, ^62^ while patients' preferences and priorities would contribute to less medical burden and more desired healthcare. Given the differences in patients' preferences, an individualized treatment option provided by physicians can motivate the practice of SDM.^1^ Furthermore, physicians emphasized that good communication skills and supporting organizational context were required to probe the patients' preferences and work out an individualized treatment option.^1^

This review found that physicians' communication skills were the most reported category in the structure dimension. If physicians are less skilled in communication, patients may be reluctant to share views, and this may also limit the opportunity for patients to receive high‐quality medical information that needs to be understood and applied in their decision.^2^ Additionally, physicians' lack of communication skills can be attributed to the absence of humanistic training.^63^ Physicians go into the practice of SDM without appreciating communication as there has been no focus on training of humanistic qualities. In addition, considering the impact of the patients' health status, personality and demographic characteristics on physician–patient communication, we recommend that targeted training should also be provided in advance for various patients.

In agreement with previous studies, we found that physicians needed supporting organizational context, including additional time, resource supply and therapeutic environments, to ensure comprehensive dialogues to share medical information from diverse disciplines and understand patient preferences during the SDM process. Consistent with other studies, decision‐making time was the main category influencing the use of SDM.^28^, ^43^ Both physicians and patients complained that due to time constraints, because of the brief consultation, sufficient explanation may not be provided, resulting in poor communication between them.^27^, ^43^ Then, the provision of sufficient resources in terms of staff and space can help distribute physicians' workloads, enabling longer interactions and provision of safe spaces for patients to express themselves without fear.^44^, ^55^ Moreover, Glyn et al.^64^ stated that the first step of the SDM model was ‘Team talk,’ with an emphasis on the importance of collaboration. In places where there is an environment for multidisciplinary cooperation, and puts a high value on patient‐centred communication, SDM is considered to be more of the norm.^51^, ^59^

Considering that the uncertainty and complexity of treatment decisions have consequences on the well‐being of patients, it is essential to motivate patients to participate in SDM.^44^ Joseph‐Williams et al.^17^ stressed the significance of knowledge for the patient when engaging in SDM, and noted that patients often have no interest in SDM, undervaluing their knowledge of personal experiences and medical information. Furthermore, better family education and higher health literacy, accompanied by higher social status, more stable employment status and strong social network relationships, all potentially indicate that wider medical knowledge can be accessed.^31^, ^33^, ^56^, ^65^ Moreover, a systematic review confirmed that SDM interventions, for example, using the Decision Aids, significantly improved medical knowledge of people with poor health literacy and lower social capital, which in turn increased their motivation to participate in SDM, perhaps more efficiently than patients with high health literacy and social capital.^66^ Decisions aids based on evidence are designed to assist patients in preparing for decision‐making by weighing the trade‐offs of treatment options.^4^ Our findings show that although Decision Aids help patients enhance medical knowledge and identify their priorities, they should still be supported with information by Decision Aids consistent with patients' developmental stage and literacy level.

Healthcare professionals, primarily physicians, are expected to guide patients through treatment options by playing a dominant role in SDM, while nurses are expected to complement and coordinate with the physicians' efforts. Some studies indicated that female and white physicians described as nice and empathetic were consistently deemed to be motivating for patients to engage in SDM.^42^ Moreover, patients' respect for physicians and positive attitudes towards SDM appear to be more significantly related to patients' motivation for SDM.^28^, ^67^ However, quite a few physicians view SDM negatively for the following reasons: First, there is only one best treatment option and involving the patient might result in poor medical treatment.^68^ Second, different views on treatment priorities of various participants are difficult to coordinate and integrate.^1^ Also, cases of emergency allow no extra time for decision communication.^50^ Third, there are unclear regulations of the additional costs incurred by SDM, and no insurance for high individual expenditure costs to the optimal treatment agreed by SDM.^69^ Based on our findings, we propose that establishing a normative standard for various contexts and settings can help allay physicians' concerns and make a case for SDM.

In the long run, SDM can meet the strategy of patient‐centred healthcare and health‐centred medical service. By promoting the participation of patients, SDM contributes to the improvement of patients' autonomy, compliance and health status, and patients have a better sense of participation in subsequent SDM practices. Similarly, physicians perform more proficiently through repeated SDM practice, resulting in efficacious use of SDM. Considering that a diagnostic procedure is not a one‐off process but a multistage service, the implementation of SDM is a cyclical and progressive process, with the current outcome facilitating better implementation in the next stage.^50^

Considering that the treatment process differs for various diseases, the decision‐making process and elements associated with SDM vary. Sorting out the factors that influence SDM in different diseases, we clustered the factors and grouped into three main themes of patient‐related, physicians‐related, and decision implementation‐related. Research focusing on SDM in the treatment of chronic diseases as well as other unspecified diseases was relatively more numerous, reported more motivation and resistance for implementing SD and focused on the distribution of factor categories across disease groups. Although the factors influencing SDM were distributed broadly overall, factors such as decision‐making time, patients' decision‐making readiness, physicians' communication skills, physicians' personal characteristics, physicians' service competency and health care environment were more concentrated in the distribution of the third and fourth types of disease treatment processes.

This systematic review has a few implications for policy and practice. Policymakers should promote SDM as a vital aspect supporting the development of a patient‐centred and value‐based healthcare system, and construct a value‐based payment system to reduce unnecessary costs. Meanwhile, a standard norm for the SDM practice is needed to guide approaches in various situations. Furthermore, the successful implementation of SDM requires sufficient support from healthcare institutions, such as additional time, private space, sensible workload and a multidisciplinary cooperation environment. In addition, communication skills, human qualities and empathy should be introduced into healthcare professionals' training curricula to use targeted interventions for different diseases. Apart from that, healthcare professionals are supposed to broaden their understanding of SDM in terms of probing for the best treatments to moving forward with the patient‐centred healthcare. In particular, physicians, who play a central role in SDM, are expected to communicate the risks of disease and treatment from the patients' perspective and empower patients to incorporate their values and preferences into the priorities of the treatment option. For patients, the use of Decision Aids to gain medical knowledge in advance would have the advantage of improving communication outcomes, reduce the decision‐making time and enable development of personalized medical options. Patients are encouraged to express their views, which differ from those of professionals, to help make medical decisions by minimizing barriers and leveraging facilitators.

## 5 LIMITATIONS

Several limitations should be noted. First, considering that the included studies include both qualitative and quantitative analyses, we calculated the frequency only for each factor; it might not indicate its magnitude. Second, the factors analysed were mostly derived from the perspectives of patients and physicians, lacking the perspective of other participants, such as nurses, healthcare institution administrators and Decisions Aids developers. Third, most included studies originated from developed regions, with less practice in LMICs. Thus, the factors summarized may not necessarily be applicable to LMICs. Considering the unique application context of each factor, we critically searched and assessed the studies, ensuring as much as possible that the emerging published research would have a negligible impact on our conclusion. Fourth, although we have elaborately categorized the factors motivating and/or leading to resistance to the implementation of SDM from the notion of four disciplines, the focus on multidisciplinary aspects in the factors is still limited, which will serve as an important aspect to guide our next in‐depth study. Finally, since the included literature was mostly published in 2016 and later, to avoid bias in the conclusions, we did not make comparisons across publication years. In addition, we did not obtain explicit evidence for age grouping of the subjects from the included literature. Therefore, we did not outline the differences in the views of participants of different age groups in this study. We will keep this limitation in mind to make our study more detailed in the future.

## 6 CONCLUSIONS

SDM achievement in practice is gratifying, but we realize that there are still numerous difficulties that hinder its widespread practice. We used the SPO model considering that it generalizes factors of motivation and resistance noted in existing studies and allows the exploration of the interactions between factors. The findings in this study indicated that, for the individual categories, decision‐making time, patients' readiness for decision‐making, physicians' communication skills and health care environment were reported more frequently. For the various themes, factor categories under the patient‐related theme showed the highest frequency and balanced distribution. An implication is that further research could usefully explore how to take a holistic approach towards patient‐related factors, and remain mindful of the changing weight in the health care environment.

## AUTHOR CONTRIBUTIONS

Changhai Tang and Anqi Wang were responsible for the conception and design of this study. Changhai Tang and Anqi Wang were responsible for evaluating and analysing the data. Changhai Tang, Anqi Wang and Jingjing Yan were responsible for drafting the manuscript, and reviewing and editing the paper. Jingjing Yan critically reviewed and commented on the draft paper. All authors have read and approved the final manuscript.

## CONFLICT OF INTEREST

The authors declare no conflict of interest.

## Supporting information

Additional File 1. PRISMA 2009 Checklist. DOC.

Additional File 2. Table S1 Information regarding the included studies. DOC.

Additional File 3. Table S2 Quality assessment of the included studies tool. DOC.

Additional File 4. References for all. DOC.

## Data Availability

The data sets used and/or analysed during the current study are available from the corresponding author on reasonable request.
