# Supplementary material for: Exploring motivations and resistances for implementing shared decision‐making in clinical practice: A systematic review based on a structure–process–outcome model
Source: Health Expect. 2022 Jun 5;25(4):1254–68. doi: 10.1111/hex.13541 (PMC9327808; doi:10.1111/hex.13541)
Supplement: Supplementary file 2 — Additional File 2. Table S1 Information regarding the included studies. DOC. [file HEX-25--s003.doc]

Table S1 Information regarding the included studies

| NO. | First Author, Year | Country | Setting | Participants | Proportion of Females | Mean age of participants (if not available: age range) | Design Study | Analysis Methods | Response Rate | Quality Score | Reporting focus: Barriers (B) and/or Facilitators (F) |
| --- | --- | --- | --- | --- | --- | --- | --- | --- | --- | --- | --- |
| 1 | Scholl, 2020 | USA | Cancer  Care | 30 diverse cancer care stakeholders (researchers, clinicians, cancer center managers, representatives of government agencies, patient, and care- giver advocates.) | 13/30 （43.33%） | 25 to 74 | semi-structured interviews | conventional content analysis | 30/36 (83.33%) | 0.85 | B & F |
| 2 | Ahmad, 2021 | Canada | Community sites, Primary care, or Specialty clinics | 18 diabetics | NR | 65 (34 to 81) | Semi-structured, one-on-one interviews | transcribed and qualitatively analyzed | 18/28 （64.29%) | 0.95 | F |
| 3 | Jordan, 2019 | UK | Adolescents living with long-term conditions | 19 adolescents | 14/19 (73.68%) | 16 (13 to 19) | participatory qualitative interviews | thematic analysis using Braun and Clarke’s  approach. | 19/66 （28.79%） | 0.85 | B & F |
| 4 | Fullwood, 2013 | UK | Chronic Disease | 29 family practices; 2 965 patients (1 372 with diabetes, 910 with COPD, 683 with IBS) | patients: 1577/2965(53.18%) | patients: 74.8±25.0 | questionnaire survey | Single-factor analysis, multi-factor analysis | family practices: 29/44 (65.91%), patients: 2965/3027(total 97.95%, DB 41%, COPD 38%, and IBS 34%） | 0.96 | B & F |
| 5 | Friedberg, 2013 | USA | Primary Care | 23 key informants, 10 patients | NR | NR | semi-structured interviews | qualitative description | 33/33 (100%) | 0.80 | B & F |
| 6 | Haimowitz, 2020 | USA | Clinical Vignettes | 319 HCPs,  290 Patients | patients: 41% | patients: 20 to 60+ | questionnaire survey | quantitative (descriptive analysis, linear regression predictive modeling, bayesian estimation, ANOVAs) and grounded theory | NR | 0.88 | F |
| 7 | Abrines-Jaume, 2016 | UK | Child and  Adolescent Mental Health Services (CAMHS) | 23 professionals (psychiatrists, psychologists, nurses, family therapists, social workers, play therapists) | NR | NR | 307 plan-do-study-act (PDSA) log books | framework analysis | NR | 0.95 | F |
| 8 | Munro, 2021 | Canada | Routine Care | 307 professionals | 77.20% | NR | cross- sectional survey, quantitative survey, open- ended survey responses | descriptive and inferential analyses, thematic analysis | 307/383 (80.16%) | 0.92 | F |
| 9 | Hamann, 2014 | Germany | Mental Healthcare | NR | NR | NR | literature search | content analysis | NR | 0.55 | B & F |
| 10 | Liverpool, 2021 | UK | Children and Young people’s Mental Health | Healthcare Professionals (HCPs): 19-interviewed, 12- in the focus group discussions, Parents: 14 interviewed, 10 in the focus groups | Parents: 22/24(91.67%) | Parents: 44.88±6.76 | focus groups, semi-structured interviews | social constructivist grounded theory | 55/55 (100%) | 0.95 | B & F |
| 11 | Peek, 2011 | USA | Community Health Centers (HC) | 974 patients with diabetes | 69% | 58±12.8 | cross-sectional survey | ordinal and logistic  regression models | 974/1483（65.68%） | 0.92 | F |
| 12 | Goto, 2021 | Japan | Primary Care | 23 physicians（SDM-Q-Doc） and 130 patients（the Japanese version of SDM-Q-9） | total 82/153(53.59%): physicians：11/23(48%),  patients:  71/130（55%） | physicians: 30s to 40s,  patients:20s to 80s | cross-sectional survey | correlation analysis, multiple regression analysis and structural equation modeling | total 153/166 (92.17%):  physicians：23/23 (100%), patients: 130/143 (90.91%) | 0.88 | F |
| 13 | Kehl, 2015 | USA | Cancer Care | 5 315 patients | 2480/5315 （46.67%） | <54 to >75 | interview and questionnaire survey | bivariable logistic regression, multivariable logistic regression | 5315/9737 (54.57%) | 1.00 | B |
| 14 | Luo, 2021 | China | Public Tertiary Hospitals in Shanghai | 2 585 inpatients | 52.19% | 55.86% (<60), 44.14% (≥60) | cross-sectional questionnaire survey | linear or two-level regression models | 2585/2585 (100%) | 1.00 | F |
| 15 | Frosch, 2012 | USA | Primary health care | 48 patients | 30/48 (62.5%) | 64.7±12.1 | focus-group sessions | constant comparative | 48/51 (94.12%) | 0.95 | B |
| 16 | Hofstede, 2013 | Netherlands | Multidisciplinary Sciatica Care | 8 general practitioners, 8 physical therapists, 8 neurologists, 8 neurosurgeons, and 8 orthopedic surgeons | total 11/40(27.5%): (3/8 general practitioners, 4/8 physical therapists, 2/8 neurologists, 2/8 neurosurgeons, and 0/8 orthopedic surgeons | general practitioners: 49, physical therapists: 47, neurologists: 49, neurosurgeons: 50, orthopedic surgeons: 52 | semi-structured interviews | directed content analysis | total 40/93 (43.01%) : neurosurgeons (8/8, 100%), orthopedic surgeons (8/10, 80%), physical therapists (8/14, 57.14%), neurologists (8/16, 50%), and general practices (8/45, 17.78%) | 0.95 | B & F |
| 17 | Hayes, 2019 | UK | Child and Youth  Mental Health | 15 clinicians | 13/15 (86.67%) | 43.11±9.65 | qualitative semi-structured  interviews and focus groups | thematic analysis, theoretical domains framework | 15/15 (100%) | 0.95 | B & F |
| 18 | Peek, 2009 | USA | African-Americans with diabetes | 51 African-Americans with diabetes | 82% | 62 | in-depth interviews and focus groups | phenomenological methodology | 51/51 (100%) | 0.85 | B & F |
| 19 | McCarter, 2016 | USA | Oncology Inpatient and Outpatient Settings | 19 oncology nurses and 11 oncology nurse practitioners | 28/30 （93.33%） | 30 to 60+ | semi-structured interviews | directed content analysis | 30/30 (100%) | 0.95 | B & F |
| 20 | Boland, 2016 | Canada | Paediatric Hospital | 57 stakeholders participated | NR | NR | focus groups and interviews | inductive thematic analysis | 57/57 (100%) | 0.90 | B & F |
| 21 | Brembo, 2020 | Norway | Routine Orthopedic Outpatient Consultations | 19 patients | 12/19(63.16%) | 40 to 84 | observe and audio record | iterative thematic approach inspired by template analysis (TA) | 19/20 (95%) | 0.95 | B & F |
| 22 | Huang, 2021 | China | Mental Healthcare | 33 mental health professionals | 18/33 (54.54%) | 33.25±9.09 | face-to-face individual interviews and focus groups | qualitative description | 33/37 （89.19%） | 0.90 | B |
| 23 | Rood, 2015 | Netherlands | Hematological Malignancies | 458 patients | 45% | 60.2 | cross-sectional survey | the Kruskal–Wallis test | 458/694 （66.99%） | 0.92 | B |
| 24 | Schapira, 2019 | USA | Breast Cancer Treatment Consultation | 14 Spanish-speaking and 32 English-speaking patients | 100% | 51 | audio-recorded | multivariate regression analysis | 46/50 (92%) | 0.92 | F |
| 25 | Rosenberg-Yunger, 2018 | Canada | Community Diabetes Management | 16 pharmacists | 10/16 (62.5%) | 46.25±12.79 | semi-structured interviews | thematic analysis | 16/19 (84.21%) | 0.85 | F |
| 26 | Roodbeen, 2020 | Netherlands | Hospital-based Palliative Care | 17 HCPs (11 physicians and 6 nurses) | 10/17 (58.82%) | NR | qualitative interview | thematic analysis following the phases described by Braun and Clarke | 17/21 （80.95%） | 0.95 | B & F |
| 27 | Clark, 2020 | Canada | Transgender Youth's Care | 47 participants: 21 trans youth, 15 parents of trans youth and 11 health care providers | NR | trans youth: 14 to 18 | semi-structured interviews | a constructivist grounded theory approach | 47/47 (100%) | 0.95 | B & F |
| 28 | Shepherd, 2014 | UK | Antipsychotic Prescribing | 26 consultant psychiatrists | NR | NR | semi-structured interview schedule（electronic communication） | interview transcripts | 26/27 (96.30%) | 0.95 | B & F |
| 29 | Hawley, 2017 | USA | Cancer Care | NR | NR | NR | NR | NR | NR | 0.55 | B & F |
| 30 | Nejati, 2019 | Iran | Multiple Myeloma | 276 patients | 133/276（48.19%) | 62.86±15.45 | questionnaire survey | descriptive analysis, structural equation model | 276/311 （88.75%） | 0.88 | B & F |
| 31 | Savelberg, 2019 | Netherlands | Breast Cancer Care | 27 clinicians: 9 breast cancer surgeons, 11 nurse practitioners and 7 nurses. | NR | NR | NR | NR | 27/34 (79.41%) | 0.95 | B |
| 32 | Henselmans, 2020 | Netherlands | Palliative Systemic Treatment | 31 oncologists and 194 patients | total 119/225(52.89%) oncologists: 24/31(74.19%); patients: 95/194(48.97%) | oncologists: 41.5±9.5, patients: 63.6±11.2 | multicenter randomized controlled trial | Ratio; Relative risk; Mixed linear models | total 225/277 (81.23%): oncologists: 31/36 (86.11%), patients: 194/241 (80.50%) | 1.00 | F |
| 33 | Kanzaria, 2015 | USA | Emergency Care | 435 emergency physicians (EPs) | 31% | 42 | questionnaire survey | descriptive analysis | 435/478 (91.00%) | 0.90 | B |
| 34 | Treffers, 2020 | Austria | Oncology Treatment Decisions | NR | NR | NR | case studies | process model | NR | 0.55 | F |
| 35 | Barton, 2014 | USA | Rheumatoid Arthritis Treatment Decisions | 509 patients | total 434/509 (85.27%):  UCSF Cohort 198/234 (84.62%),  RA Panel 236/275 (85.82%) | UCSF Cohort: 55±14,  RA Panel: 64±11 | cohort study （a one-time telephone interview administered to 2 separate cohorts of adults with RA） | descriptive analysis, unadjusted and adjusted logistic regression models | NR | 0.88 | B |
| 36 | Maes-Carballo, 2021 | Spain | Breast Cancer (BC) Treatment | 459 doctors | 238/456(52.19%) | age groups | cross-sectional study based on an online questionnaire | Chi-square test, Student t-test, ANOVA | 459/541 (84.84%) | 0.88 | B |
| 37 | Manhas, 2020 | Canada | Community Rehabilitation | 49 participants: 23 patients, 26 providers | total 36/49 (73.45%): patients 17/23 (73.91%) and providers 19/26 (73.08%). | NR | semi-structured interviews, convenience sampling | contents analysis | 49/49 (100%) | 1.00 | B & F |
| 38 | Vaillancourt, 2015 | Canada | Dyslipidemia Nutrition Therapy Consultation | 13 dietitians; 13 patients | total 19/26 (73.08%):  dietitians: 13/13 (100%) patients: 6/13 (46.15%) | Dietitians: 38.9±10.8 patients: 53.8±14.6 | questionnaire survey | descriptive statistics, Spearman’s partial correlations | total 26/39 (66.67%) dietitians: 13/26 (50%) patients: 13/13 (100%) | 0.88 | B & F |
| 39 | Desroches, 2011 | Canada | Behavioral Beliefs of Dietitians | 21 dietitians | 100% | 39.3±11.3 | semi-structured interview | thematic content analysis | 21/40 (52.5%) | 1.00 | B & F |
| 40 | Mathijssen, 2020 | Netherlands | Rheumatology | 77 physicians,  70 nurses | total 120/147(81.63%): physicians: 51/77, nurses: 69/70 | physicians: 45.7±9.5,  nurses 50.7±9.0 | cross- sectional, exploratory, online survey | descriptive analysis, t- test or Fisher’s exact test | total 147/705 (20.85%): physicians: 77/454 (16.85%), nurses: 70/251 (27.89%) | 0.88 | B & F |
| 41 | Bisset, 2020 | UK | Colorectal Surgeons | 296 patients | 72% | 55.1% aged 40–59 years | online survey, a face-to-face patient and public involvement (PPI) exercise | Quantitative analysis, Thematic analysis | 296/2063 (14.35%) | 0.79 | F |
| 42 | Lee, 2020 | Malaysia | Primary  Care Consultations | 199 patients and 31 doctors | total 125/230 (54.35%): patients: 105/199 (52.76%) doctors: 20/31 (64.52%) | Patients: 57.5 doctors: 33.0 | cross-sectional survey | univariate analysis, multiple linear regression model | NR | 0.88 | F |
| 43 | Menear, 2018 | Canada | Primary Care Physicians | 114 eligible licensed primary care physicians | 64/114 (56.14%) | 45.3±9.5 | the EXACKTE^2^ study: a multicenter cross- sectional survey which enrolled large number of unique patient- physician dyads | descriptive analyses, normality test, intraclass correlation, multivariate linear regression models | 114/170 (67.06%) | 1.00 | F |
| 44 | Chang, 2019 | China，T aiwan | Cancer Patient Care | 120 patients | 60/120 （50%） | 54.5±12.0 | A cross-sectional descriptive study | Student t-test, ANOVA, Structural equation modeling | 120/120 (100%) | 0.92 | F |
| 45 | Smalley, 2014 | USA | Children with Special Health Care Needs | Children: unweighted 40 242, weighted 11 102 | 20542/51344 (40.01%）：unweighted 16033/40242 (39.84%), weighted 4509/11102 (40.61%) | 0 to 17 | 2009-2010 NS-CSHCN | Multiple logistic regression | Over two thirds | 0.92 | B |
| 46 | Wang, 2019 | China，T aiwan | Glycemic Control in Type 2 Diabetes | 372 eligible patients | 227/372 (61.02%) | Age groups | cross-sectional survey （self-administered questionnaires ） | descriptive statistics, multiple regression analysis | 372/400 (93%) | 0.92 | F |
| 47 | Wigfall, 2018 | USA | Healthcare Engagement | 1 604 information seekers | 913/1604 (56.92%) | <50: 942/1604(58.72%), ≥50: 662/1604(41.27%) | cross-sectional study, data from the third cycle of the fourth iteration of the Health Information National Trends Survey | multivariate logistic regression analyses | 1604/3185 (50.36%) | 0.96 | F |
| 48 | Solberg, 2014 | USA | Usual Primary Care | 1 168patients | 851/1168（72.86%） | 44.2 | questionnaire survey | univariate& multivariate general linear model | 1168/7155 (16.32%) | 0.96 | B & F |
| 49 | Schneider, 2006 | Germany | Primary Healthcare | 234 GPs patients | 62% | female: 44.74±16.08,  male: 42.96±18.08 | questionnaire survey | descriptive analysis, ANOVA | 234/358 (65.36%) | 0.92 | B & F |
| 50 | Holmes-Rovner, 2000 | USA | Fee-for-Service Hospital Systems | 34（14 physicians, 13 nurses, 7 social workers and administrators | NR | NR | questionnaire survey, participant observation | descriptive analysis | 34/34（100%） | 0.96 | B |
| 51 | Paredes, 2018 | USA | Medical Expenditure Panel Survey | 13 880 patients | 6605/13880 (47.59%) | Age groups | Medical Expenditure Panel Survey database | descriptive analysis, multiple regression analysis | 13880/71815（19.33%） | 0.88 | B |
| 52 | Koizumi, 2018 | Japan | Oncofertility Treatment | 33 female patients,  19 family members | female patients: 33/33(100%), family members: NR | NR | observational, cross-sectional study | chi-square | total 52/52 (100%): patients 33/33 (100%), family members 19/19 (100%) | 0.75 | B |
| 53 | Bot, 2014 | USA | Massachusetts General Hospital | 130 patients | 68/130(52.31%) | 52±16 | questionnaire survey | Pearson correlations, t-tests, one-way analysis of variance, regressions | 130/145 (89.66%) | 0.92 | B & F |
| 54 | Bradley, 2018 | UK | Adult Mental Health Care | 46 family members, 55 staffs | family members: 30/46(65.22%), staffs: unknown | family members: 18 to 80,  staffs: not report | open- ended questions | thematically analysis | family members: not possible to estimate, staffs: 55/158 (34.81%) | 0.90 | B & F |
| 55 | Xu, 2017 | China | Public Specialist Outpatient Clinics | 13 966 patients | 8030/ 13966（57.50%） | 18 to 61+ | territory-wide cross-sectional telephone survey | description; ANOVA, logistic regression models | 13966/22525（62.00%） | 1.00 | B & F |
| 56 | Stalnikowicz, 2020 | Israeli | NR | NR | NR | NR | literature search | narrative review of the literature | NR | 0.70 | B & F |
| 57 | Hofstede, 2014 | Netherlands | Hospital Care | 246 professionals (general practitioners, physical therapists, neurologists, neurosurgeons, orthopedic surgeons), 155 patients | total 160/401 (39.90%): professionals: 73/246 (29.67%), patients: 87/155 (56.13%) | professionals: 46±10.0,  patients: 50±13.2 | questionnaire survey | descriptive statistics, independent t-test, Mann Whitney U, Fisher’s exact or chi-square tests | total 401/442 (90.72%): professionals: 246/280 (87.86%), patients: 155/162 (95.68%) | 1.00 | F |
| 58 | Sonntag, 2012 | Germany | Primary Care | 10GPs | 7/10(70%) | 51 | audiotape the individual | chi-square test, Student’s t test analysis | 10/12 (83.33%) | 0.92 | B & F |
| 59 | Ousseine, 2019 | France | Cancer Assistance Research | 2 299 (cancer patients/survivors (primarily breast) and/or other people) | 2223/2299 (96.69%) | 47.7±13.6 | cross-sectional survey | binary logistic regression models | 2299/2568 (89.52%) | 0.96 | B & F |
| 60 | Berger-Hoger, 2019 | Germany | Certified Breast Care Centers in Germany. | 50 physicians, 31 nurses | total 69/81 (85.19%): physicians: 38/50 (76%), nurses: 31/31 (100%) | Physicians (intervention group 44.6±7.7, control group 41.3±9.7), nurses (inter group 47.7±8.5, control group 53.2±3.2) | a multi-center superiority cluster randomized controlled trial | descriptive statistics, cluster analysis, t-tests, non-parametric Wilcoxon test | total 81/84 (96.43%)  physicians：50/53 (94.33%), patients: 31/31 (100%) | 0.93 | B & F |
| 61 | Tam-Seto, 2015 | Canada | Adolescent Mental Health Occupational Therapy | 6 therapists | NR | NR | telephone interview | content analysis | 6/6(100%) | 1.00 | B & F |
| 62 | Butterworth, 2014 | UK | GP Surgeries | 20 patients | 11/20(55%) | ≥65 | semi-structured interview | qualitative description | 20/50(40%) | 0.90 | B & F |
| 63 | Tariman, 2016 | USA | Oncology Nursing and Cancer Treatment | 19 oncology nurses and 11 oncology nurse practitioners | majority | 40 to 59 | Semi-structured interviews | content analysis | 30/30 (100%) | 0.90 | F |
| 64 | Lin, 2020 | China | Mental Healthcare | 20 patients | 7/20 (35%） | 38 | semi-structured interviews | thematic analysis | 20/92 (21.74%) | 1.00 | B & F |
| 65 | Evong, 2019 | Canada | Pediatric Otolaryngology Surgical Consultations | 117 parents | 92/117 (78.63%) | female: 33.16±5.0, male: 35.33±6.1 | questionnaire | Description, Spearman’s correlation coefficient | 117/131 (89.31%) | 0.96 | F |
| 66 | Schoenfeld, 2019 | USA | Emergency  Department | 15 emergency physicians | 6/15 (40%） | 46 | Semi-structured interviews | qualitative content analysis | 15/15 (100%) | 1.00 | B & F |
| 67 | Davis, 2011 | USA | Primary Care | academic clinicians (n=16), interns/residents (n=84) and community clinicians (n=35). | total 71/135(52.59%): (academic clinicians 10/16, interns/residents 42/84, community clinicians 19/35) | total 36.1±10.3 (academic clinicians 41.6±8.1, interns/residents 22.9±3.2, community clinicians 48.6±9.6) | questionnaire survey | descriptive analysis | total 135/152 (88.82%): academic clinicians 16/18 (88.89%), interns/residents 84/84 (100%), community clinicians 35/50 (70%) | 0.96 | B |
| 68 | Padilla Garrido, 2019 | Spain | Oncology Treatment Practice | 130 physicians | 63/130 (48.46%) | 45.6 | questionnaire survey | descriptive analysis, χ^2^ association test, the Wilcoxon signed rank test, the Kruskal-Wallis H test, and the Mann-Whitney U test | 130/351(37.04%) | 0.96 | B & F |
| 69 | Zeuner, 2015 | USA | Academic Medical Centre | 20 physicians | 13/20（65%） | NR | semi-structured qualitative interviews | Fishbein’s Integrative Model of Behavior Prediction | 20/22 (90.91%) | 0.90 | B |
| 70 | Young, 2008 | USA | Primary Depression Care | 18 standardized patients (SPs), 151 physicians | physicians:33% | 46.1±9.8 | observation method | description statistics, partial correlation analyses, generalized linear mixed models | total 169/170 (99.41%): SPs 18/18 (100%), physicians 151/152 (99.34%) | 0.96 | B & F |
| 71 | Fukui, 2014 | USA | Psychiatric Care | 128 patients | 65/128 (50.78%) | 43.4±10.63 | manual scoring | multilevel linear regression analysis | 128/170 (75.29%) | 0.92 | F |
| 72 | van den Brink-Muinen, 2011 | Netherlands | Chronically Ill and Disabled Patients Care | 812 chronically ill and disabled patients | 68% | 60±15 | questionnaire survey | chi-square tests, Multilevel regression analyses | 812/2423 (33.51%) | 1.00 | F |
| 73 | Spies, 2006 | Germany | Anesthesiological Settings | 341 patients (151 patients of the premedication visit, 190 chronic pain patients) | total 190/341 (55.72%): premedication: 63/151 (41.72%), chronic pain patients: 127/190 (66.84%) | premedication: 48.4±17.4,  chronic pain patients: 57.4±11.6 | questionnaire survey | description statistics; χ^2^-test, the student’s t-test, the Mann–Whitney U-test, Multiple linear regression analysis | total 341/584 (58.39%): premedication: 151/280 (53.93%), chronic pain patients: 190/304 (62.5%) | 0.96 | F |
| 74 | Baig, 2020 | Pakistan | Diabetes Management | 11 consultants | NR | NR | in-depth interviews | grounded theory | 11/14 (78.57%) | 0.95 | B & F |
| 75 | Peek, 2010 | USA | Race and SDM | 51 patients (24 in depth Interviews, 27 in focus groups) | 82% (depth Interviews: 83%, focus groups: 81%) | depth Interviews: 66, focus groups: 59 | semi-structured interviews and focus groups | content analysis | total 51/83 (61.45%): interview: 24/36 (66.66%); focus group: 27/47 (57.45%) | 1.00 | B |
| 76 | Charles, 2004 | Canada | Breast Cancer | 232 surgeons, 102 oncologists | total 54/324 (16.67%): Surgeons: 20/226 (8.85%), Oncologists: 34/100 (34.0%) | Age groups | cross-sectional survey, focus groups | Descriptive analysis | total 334/452 (73.89%): surgeons 232/322 (72.04%), oncologists 102/130 (78.46%) | 0.96 | B & F |
| 77 | Hamann, 2017 | Germany | Mental Health Services | 329 patients: 126 schizophrenia spectrum disorder, 203 affective disorder | 187/329 (56.84%) | 43.36±12.9 | questionnaire | Linear Regression Analyses, Structural Equation Approach | 329/329 (100%) | 0.96 | B & F |
| 78 | Barker, 2019 | Canada | Antenatal Counselling in Anticipated Extremely Preterm Birth | 25 health care providers | 19/25 (76%） | NR | semi-structured interviews | qualitative descriptive, content analysis approach | 25/55 (45.45%) | 0.90 | B & F |
| 79 | Ashoorian, 2021 | Australia | Mental Health Services | NR | NR | NR | literature research | content analysis | NR | 0.75 | B & F |
| 80 | Mahone, 2011 | USA | Mental Health Treatment | 44（family members (n=4), prescribers (psychiatrists and psychiatric nurse practitioners) (n= 4), other providers (nurses, case managers, support staff) (n=6), rural clinic staff (n=6) and three consumer groups (n=8,7,9). ） | 66% | NR | participatory-action research, focus groups | content analysis | NR | 1.00 | B |
| 81 | Bouma, 2014 | USA | Safety Net in Primary Care | 15 clinicians | NR | NR | questionnaire survey, semi-structured interview | theme analysis | 15/23 (65.22%) | 0.80 | B |
| 82 | Lamb, 2019 | USA | Physician's Decision‐Making Style | 330 physicians | 86/330 (26.06%) | NR | questionnaire survey | factor analyses, Chi‐Squared, Structural equation | 330/350 (94.29%) | 0.92 | F |
| 83 | Moleman, 2020 | Netherlands | Medical Disciplines | 68 healthcare professionals, 15 patients | NR | NR | semi-structured interviews | content analysis | professionals: 68/83 (81.93%), patients: 15/15 (100%) | 1.00 | B |
| 84 | Brogan, 2018 | UK | Health and Social Care Trust | 43 healthcare providers (HPs) | 35/43(81.40%) | 47 | focus groups | thematical analysis | 43/54 (79.63%) | 0.95 | B |
| 85 | Vedasto, 2021 | Tanzania | Tertiary Hospital diabetic Clinic | 11（4 Healthcare providers, 7 patients) | total 5/11 (45.45%): Healthcare providers 2/4 (50%), patients: 3/7 (42.86%) | physicians:30s to 40s, patients: 46 to76 | face-to-face in-depth interview | inductive content analysis | 11/18 (61.11%) | 0.95 | B |
| 86 | Suurmond, 2006 | Netherlands | Intercultural Context | 18 physicians, 13 immigrant patients | NR | patients: 20 to 78 | Literature, semi-structured interview | exploratory and descriptive analysis, content analysis | 31/31 (100%) | 0.85 | B |
| 87 | Mariani, 2017 | Italy and the Netherlands | Dementia Care | 19 healthcare professionals (9 The Netherlands, 10 Italy) | 16/19 (84.21%) | The Netherlands: 41, Italy: 49.5 | literature review, semi-structured interview, focus group interviews | content analysis | 19/19 (100%) | 1.00 | B & F |
| 88 | Acerini, 2018 | UK | Growth Hormone Therapy | NR | NR | NR | NR | NR | NR | 0.60 | B & F |
| 89 | Miron-Shatz, 2012 | Israeli | Healthcare System | NR | NR | NR | NR | NR | NR | 0.60 | B |
| 90 | Hoang, 2020 | USA | Chronic Medical Conditions | 27 parents, 27 children, 16 faculty | total: 40/70(57.14%): parents: 16/27(59.26%), children: 12/27(44.44%), faculty: 12/16(75%) | parents:20 to 49, children:0 to 19, faculty: 30 to 49 | Semi-structured interviews | modified grounded theory | 80/80 (100%) | 0.90 | B |
| 91 | Rose, 2017 | UK | Rehabilitation Centre and Patients’ Homes | 19 rehabilitation team members and 40 patients | NR | patients: 83 | mixed-methods approach: questionnaires and semi- structured interviews | Cross-tabulations, descriptively analyzed, Welch test, Games-Howell tests, thematic analysis | Total 59/89 (66.29%): physicians: 19/24 (79.17%); patients: 40/65 (61.54%) | 0.95 | B & F |
| 92 | Renz, 2013 | USA | Multi-specialty, Fee-for-Service Clinical Settings | 15 stakeholders (3 health system leaders, 3 practicing providers, 3 SDM implementation project managers, 2 medical malpractice liability insurers, and 4health plan leaders) | NR | NR | Semi-structured interviews | thematic analysis | NR | 0.90 | B & F |
| 93 | Peek, 2014 | USA | Primary Care Visits | 50 physicians, 273 patients | patients:66.1% | patients:58.3±13.2 | questionnaire survey | descriptive analysis, multivariable mixed linear and logistic regression | patients: 273/279 (97.85%) | 0.89 | F |
| 94 | Kraaij, 2020 | Netherlands | Dermatological Diagnosis and Treatment | 219 patients, 147 physicians(dermatologists) | total 258/366(70.49%): patients: 156/219 (71.23%), Dermatologists 102/147 (69.39%) | Age groups | two comparable online surveys | Mann-Whitney U, Somers’s d test, descriptive analysis | total 376/927 (40.56%): patients 219/226 (96.90%), physicians 147/701 (20.97%) | 0.96 | B & F |
| 95 | Berger, 2012 | Germany | Different Approaches of Medicine | 20 GPs, 10 NMP (nonmedical practitioners) | 19/30(63.33%) | COM-GP: 55.7±6.7, CAM-GP: 58.0±4.7, CAM-NMP: 45.7±7.8 | interviews | qualitative analysis | 30/64 (46.88%) | 1.00 | B |
| 96 | Nijhuis, 2019 | Netherlands | Shared Decision-Making (SDM) in Advanced Parkinson | 111 Dutch persons with Parkinson | 43/111 (38.74%) | 65 | questionnaire survey | descriptive analysis, Pearson’s χ^2^ test or Fisher exact test | 111/180 (61.67%) | 0.92 | B & F |
| 97 | van Til, 2010 | Netherlands | Shared Decision-Making and Decision Aids in Rehabilitation Medicine | 126 PRM (physical and rehabilitation medicine) physicians | 56/126 (44.44%) | 31 to 50+ | cross-sectional survey | descriptive analysis, Spearman correlations analysis | 126/408 (30.88%) | 0.96 | B & F |
| 98 | Shen, 2019 | China | Breast Cancer | 511 breast cancer patients | 511/511(100%) | 57.9±11.3 | cross-sectional survey | description analysis, Sequential linear regression models | 511/511 (100%) | 0.92 | B & F |
| 99 | Smith, 2016 | USA | Patient Activation in Preferences for Shared decision-making | 3 400 patients | 2018/3400（59.35%） | 18 to 80+ | nationally representative survey | complex samples analysis using the weighting variable | 3400/3400 (100%) | 0.92 | B |
| 100 | White, 2007 | USA | Shared decision-making at the End of Life in Intensive Care Units | 51 eligible family conferences (51 patients, 169 family members), 35 physicians | total 139/255 (54.51%): patients 26/51 (50.98%), family members 101/169 (59.76%), physicians 12/35 (34.29%) | patients: 60.0±20.3, family members: 48.0±15.8, physicians: 38.0±9.5 | family conferences | mixed-effects regression model | total 86/146 (58.90%): eligible family conferences: 51/111 (45.95%), physicians: 35/35 (100%) | 0.96 | F |
| 101 | Pellerin, 2011 | Canada | Family Medicine | 152 patients | 106/152 (69.73%) | NR | the observing patient involvement in decision-making (OPTION) scale | descriptive and inferential statistics | 152/159 (95.60%) | 1.00 | F |
| 102 | Hamann, 2016 | Germany | Acute Mental Health Settings | 7 focus groups (16 patients, 17 physicians) | total 14/33 (42.42%): patients: 8/16 (50%), physicians: 6/17 (35.29%) | patients: 41.8±14.6; physicians: 44.9±7.7 | focus groups | content analysis | 33/33(100%) | 1.00 | B & F |
| 103 | Keij, 2021 | Netherlands | Disease Treatment | 15 patients, 16 professionals (6 physicians, 3 nurses, 2 general practitioners, 5 researchers) | totals 22/31 (70.97%): patients: 11/15 (73.33%), professionals 11/16 (68.75%) | patients: 69, physicians: 42, nurses: 55, general practitioners: 48.5, researcher: not report | semi-structured interviews | Grounded Theory approach | patients: 15/17 (88.24%) | 1.00 | B & F |
| 104 | Luciano, 2020 | Germany | Psychiatric Clinical Routine Mental Health Care | 213 professionals (psychiatrists, nurses, support workers, social workers, or occupational therapists), 563 patients | total 435/776 (56.06%): professionals: 128/213 (60.09%), patients: 307/563 (54.53%) | professionals: 45.9±10.5,  patients: 41.7±10.7 | questionnaire survey | multivariable logistic models | patients: 563/588 (95.75%) | 0.92 | B & F |
| 105 | Astbury, 2017 | UK | Health Visitor Practice | 9 health visitors, 9 parents | NR | NR | semi-structured interviews, questionnaire | content analysis | 100% | 1.00 | B & F |
