# Supplementary material for: Exploring motivations and resistances for implementing shared decision‐making in clinical practice: A systematic review based on a structure–process–outcome model
Source: Health Expect. 2022 Jun 5;25(4):1254–68. doi: 10.1111/hex.13541 (PMC9327808; doi:10.1111/hex.13541)
Supplement: Supplementary file 3 — Additional File 3. Table S2 Quality assessment of the included studies tool. DOC. [file HEX-25--s002.doc]

Table S2 Quality assessment of the included studies

| Criteria for quantitative studies | Agreement (%) | Disagreement (%) |  | Criteria for qualitative studies | Agreement (%) | Disagreement (%) |
| --- | --- | --- | --- | --- | --- | --- |
| Question / objective sufficiently described? | 52 (100) | 0 |  | Question / objective clearly described? | 51 (96.23) | 2 (3.77) |
| Design evident and appropriate to answer study question? | 42 (80.77) | 10 (19.23) |  | Design evident and appropriate to answer study question? | 48 (90.57) | 5 (9.43) |
| Method of subject selection / source of information / input variables appropriately described? | 44 (84.62) | 8 (15.38) |  | Context for the study clear? | 49 (92.45) | 4 (7.55) |
| Subject characteristics or input variables / information sufficiently described? | 46 (88.46) | 6 (11.54) |  | Connection to a theoretical framework / wider body of knowledge? | 41 (77.36) | 12 (22.64) |
| If random allocation to treatment group was possible, is it described? | 40 (76.92) | 12 (23.08) |  | Sampling strategy relevantly and fairly described? | 47 (88.64) | 6 (11.32) |
| If interventional and blinding of investigators to intervention was possible, is it reported? | 52(100.00) | 0 (0.00) |  | Data collection methods systematic, and clearly described? | 51 (96.23) | 2 (3.77) |
| If interventional and blinding of subjects to intervention was possible, is it reported? | 52(100.00) | 0 (0.00) |  | Data analysis complete and systematic, and clearly described? | 53 (100) | 0 (0.00) |
| Outcome and exposure measure(s) well defined and robust to measurement / misclassification bias? Means of assessment reported? | 49 (94.23) | 3 (5.77) |  | Use of verification procedure(s) to establish credibility of the study? | 48 (90.57) | 5 (9.43) |
| Sample size appropriate? | 47 (90.38) | 5 (9.62) |  | Conclusions supported by the results? | 52 (98.11) | 1 (1.89) |
| Analysis described and appropriate? | 49 (94.23) | 3 (5.77) |  | Reflexivity of the account? | 40 (75.47) | 13 (24.53) |
| Some estimate of variance is reported for the main results/outcomes? | 47 (90.38) | 5 (9.62) |  |  |  |  |
| Controlled for confounding? | 38 (73.08) | 14 (26.92) |  |  |  |  |
| Results reported in sufficient details? | 48 (92.31) | 4 (7.69) |  |  |  |  |
| Do the results support the conclusions? | 50 (96.15) | 2 (3.85) |  |  |  |  |

Note: Assessing quality of the included studies by QualSyst tool
