# Supplementary material for: Exploring motivations and resistances for implementing shared decision‐making in clinical practice: A systematic review based on a structure–process–outcome model
Source: Health Expect. 2022 Jun 5;25(4):1254–68. doi: 10.1111/hex.13541 (PMC9327808; doi:10.1111/hex.13541)
Supplement: Supplementary file 4 — Additional File 4. References for all. DOC. [file HEX-25--s004.doc]

**References**

1. MJ Barry S E. Shared Decision Making—The Pinnacle of Patient-Centered Care. *New Engl J Med*. 2012;366(9):780-781

2. Bomhof-Roordink H, Grtner FR, Stiggelbout AM, Pieterse AH. Key components of shared decision making models: a systematic review. *BMJ Open*. 2019;9(12): e031763

3. Goto Y, Miura H, Son D et al. Association between physicians' and patients' perspectives of shared decision making in primary care settings in Japan: The impact of environmental factors. *PLoS One*. 2021;16(2): e0246518

4. Jennifer, Freytag. Barriers that define a genre of shared decision making in palliative care communication. *Journal of Communication in Healthcare*. 2012;5(2):140-146

5. James K, Quirk A. The rationale for shared decision making in mental health care: a systematic review of academic discourse. *Mental Health Review Journal*. 2017;22(3):1-23

6. Pollard S, Bansback N, Bryan S. Physician attitudes toward shared decision making: A systematic review. *Patient Education & Counseling*. 2015;98(9):1046-1057

7. Dirmaier IS, Loon MK, Sepucha K et al. Measurement of shared decision making - a review of instruments. *Zeitschrift Fuer Evidenz Fortbildung Und Qualitaet Im Gesundheitswesen*. 2011;105(4):313-324

8. Worral MTJT. A qualitative systematic review of internal and external influences on shared decision-making in all health care settings. *JBI Database of Systematic Reviews and Implementation Reports*. 2014;12(5):121-194

9. Covvey JR, Kamal KM, Gorse EE, Mehta Z, Zacker C. Barriers and facilitators to shared decision-making in oncology: a systematic review of the literature. *Support Care Cancer*. 2019;27(9):1613-1637

10. Braddock CH. The emerging importance and relevance of shared decision making to clinical practice. *Medical Decision Making*. 2010;30(5 Suppl):5S-7S

11. Coulter A, Edwards A, Elwyn G, Thomson R. Implementing shared decision making in the NHS. *BMJ: British Medical Journal* (Overseas & Retired Doctors Edition). 2010;341(4):971-973

12. H Rter M, Müller H, Dirmaier J et al. Patient participation and shared decision making in Germany - history, agents and current transfer to practice. *Ztschrift Für Evidenz Fortbildung Und Qualitt Im Gesundhtswesen*. 2011;105(4):263-270

13. Pel-Littel RE, Snaterse M, Teppich NM, Buurman BM, Reimer W. Barriers and facilitators for shared decision making in older patients with multiple chronic conditions: a systematic review. *BMC Geriatrics*. 2021;21(1):112-125

14. Boland L, Graham ID, Légaré F, Lewis K, Stacey D. Barriers and facilitators of pediatric shared decision-making: A systematic review. *Implement Sci*. 2019;14(1):7-31

15. Joseph-Williams N, Elwyn G, Edwards A. Knowledge is not power for patients: A systematic review and thematic synthesis of patient-reported barriers and facilitators to shared decision making. *Patient Education & Counseling*. 2014;94(3):291-309

16. Castillo H, Ramon S. "Work with me": service users' perspectives on shared decision making in mental health. *Mental Health Review Journal*. 2017;22(4):1-32

17. Malone H, Biggar S, Javadpour S, Edworthy Z, Coyne I. Interventions for promoting participation in shared decision-making for children and adolescents with cystic fibrosis: *Protocols*. 2017;

18. Donabedian A. Evaluating the quality of medical care. *Milbank Q*. 2010;83(3):691-729

19. Mahdavi M, Vissers J, Elkhuizen S, Dijk MV, Klundert J. The relationship between context, structure, and processes with outcomes of 6 regional diabetes networks in Europe. *Plos One*. 2018;13(2): e0192599

20. Whitlock LSDM. Preferred reporting items for systematic review and meta-analysis protocols (PRISMA-P) 2015: elaboration and explanation. *BMJ Clinical Research*. 2015;349

21. Higgins J, Green S. *Cochrane Handbook for Systematic Reviews of Interventions, Version 5.1.0*. The Cochrane Collaboration. The Cochrane Collaboration. 2013;

22. Kmet LM, Lee RC, Cook LS. Standard Quality Assessment Criteria for Evaluating Primary Research Papers from a Variety of Fields*.* *Health Technology Assessment Alberta Heritage Foundation for Medical Research*; 2004:1-28

23. Jesus TS, Hoenig H. Postacute rehabilitation quality of care: toward a shared conceptual framework. *Archives of Physical Medicine & Rehabilitation*. 2015;96(5):960-969

24. Huang CM, Lam L, Zhong YP, Plummer V, Cross W. Chinese mental health professionals' perceptions of shared decision-making regarding people diagnosed with schizophrenia: A qualitative study. *Int J Ment Health Nu*. 2021;30(1):189-199

25. Hofstede SN, Marang-van De Mheen PJ, Wentink MM et al. Barriers and facilitators to implement shared decision making in multidisciplinary sciatica care: a qualitative study. *Implement Sci.* 2013; 8:95-105

26. Rood JAJ, Van Zuuren FJ, Stam F et al. Cognitive coping style (monitoring and blunting) and the need for information, information satisfaction and shared decision making among patients with haematological malignancies. *Psycho‐Oncology*. 2015;24(5):564-571

27. White DB, Braddock CHR, Bereknyei S, Curtis JR. Toward shared decision making at the end of life in intensive care units: opportunities for improvement. *Archives of Internal Medicine*. 2007;167(5):461-467

28. Smalley LP, Kenney MK, Denboba D, Strickland B. Family perceptions of shared decision-making with health care providers: results of the National Survey of Children With Special Health Care Needs, 2009-2010. *Matern Child Health J*. 2014;18(6):1316-27

29. Brembo EA, Eide H, Lauritzen M, van Dulmen S, Kasper J. Building ground for didactics in a patient decision aid for hip osteoarthritis. Exploring patient-related barriers and facilitators towards shared decision-making. *Patient Educ Couns*. 2020;103(7):1343-1350

30. Chang HL, Li FS, Lin CF. Factors Influencing Implementation of Shared Medical Decision Making In Patients With Cancer. *Patient preference and adherence*. 2019; 13:1995-2005

31. Xu RH, Wong ELY. Involvement in shared decision-making for patients in public specialist outpatient clinics in Hong Kong. *Patient preference and adherence*. 2017; 11:505-512

32. Treffers T, Putora PM. Emotions as Social Information in Shared Decision-Making in Oncology. *Oncology*. 2020;98(6):430-437

33. Desroches S, Lapointe A, Deschênes S, Gagnon M, Légaré F. Exploring dietitians' salient beliefs about shared decision-making behaviors. *Implement Sci*. 2011; 6:57-57

34. Peek ME, Wilson SC, Gorawara-Bhat R et al. Barriers and facilitators to shared decision-making among African-Americans with diabetes. *J Gen Intern Med*. 2009;24(10):1135-1139

35. Solberg LI, Crain AL, Rubenstein L et al. How Much Shared Decision Making Occurs in Usual Primary Care of Depression? *Journal of the American Board of Family Medicine*. 2014;27(2):199-208

36. Lee YK, Chor YY, Tan M et al. Factors associated with level of shared decision making in Malaysian primary care consultations. *Patient Educ Couns*. 2020;103(5):1049-1051

37. Paredes AZ, Idrees JJ, Beal EW et al. Influence of English proficiency on patient-provider communication and shared decision-making. *Surgery*. 2018;163(6):1220-1225

38. Rose A, Rosewilliam S, Soundy A. Shared decision making within goal setting in rehabilitation settings: a mixed-methods study. *Patient Education & Counseling.* 2017;100(1):65-75

39. Ahmad T, Hari S, Cleary D, Yu C. "I Had Nobody to Represent Me": How Perceptions of Diabetes Health-Care Providers' Age, Gender and Ethnicity Impact Shared Decision-Making in Adults with Type 1 and Type 2 Diabetes. *Can J Diabetes*. 2021;45(1):78-88.e2

40. Hayes D, Edbrooke-Childs J, Town R, Wolpert M, Midgley N. Barriers and facilitators to shared decision making in child and youth mental health: clinician perspectives using the Theoretical Domains Framework. *Eur Child Adolesc Psychiatry*. 2019;28(5):655-666

41. Lin C, Renwick L, Lovell K. Patients' perspectives on shared decision making in secondary mental healthcare in Taiwan: A qualitative study. *Patient Educ Couns.* 2020;103(12):2565-2570

42. Friedberg MW, Van Busum K, Wexler R, Bowen M, Schneider EC. A demonstration of shared decision making in primary care highlights barriers to adoption and potential remedies. *Health Affairs*. 2013;32(2):268-275

43. Manhas KP, Olson K, Churchill K, Vohra S, Wasylak T. Experiences of shared decision-making in community rehabilitation: a focused ethnography. *Bmc Health Serv Res.* 2020;20(1):329-340

44. Padilla Garrido N, Aguado Correa F, Bayo Lozano E, Bayo Calero J, Ortega Moreno M. Physicians' awareness and assessment of shared decision making in oncology practice. *Rev Esp Salud Pública*. 2019; 93:1-12

45. Scholl I, Kobrin S, Elwyn G. "All about the money?" A qualitative interview study examining organizational- and system-level characteristics that promote or hinder shared decision-making in cancer care in the United States. *Implement Sci.* 2020;15(1):81-89

46. Nijhuis FAP, van den Heuvel L, Bloem BR, Post B, Meinders MJ. The Patient's Perspective on Shared Decision-Making in Advanced Parkinson's Disease: A Cross-Sectional Survey Study. *Front Neurol*. 2019; 10: 896-904

47. Mathijssen EGE, van den Bemt BJF, Wielsma S, van den Hoogen FHJ, Vriezekolk JE. Exploring healthcare professionals' knowledge, attitudes and experiences of shared decision making in rheumatology. *RMD Open*. 2020;6(1): e001121

48. McCarter SP, Tariman JD, Spawn N et al. Barriers and Promoters to Participation in the Era of Shared Treatment Decision-Making. *Western J Nurs Res*. 2016;38(10):1282-1297

49. Kanzaria HK, Brook RH, Probst MA et al. Emergency physician perceptions of shared decision-making. *Acad Emerg Med*. 2015;22(4):399-405

50. Lamb CC, Wang YM, Lyytinen K. Shared decision making: Does a physician's decision-making style affect patient participation in treatment choices for primary immunodeficiency? *J Eval Clin Pract.* 2019;25(6):1102-1110

51. Renz AD, Conrad DA, Watts CA. Stakeholder perspectives on the implementation of shared decision making: a qualitative data analysis. *Journal of Management & Marketing in Healthcare*. 2013;6(2):122-131

52. Charles C, Gafni A, Whelan T. Self-reported use of shared decision-making among breast cancer specialists and perceived barriers and facilitators to implementing this approach. *Health Expect.* 2004;7(4):338-348

53. Zeuner R, Frosch DL, Kuzemchak MD, Politi MC. Physicians' perceptions of shared decision-making behaviours: a qualitative study demonstrating the continued chasm between aspirations and clinical practice. *Health Expect*. 2015;18(6):2465-76

54. Menear M, Garvelink MM, Adekpedjou R et al. Factors associated with shared decision making among primary care physicians: Findings from a multicentre cross-sectional study. *Health Expect.* 2018;21(1):212-221

55. Ashoorian DM, Davidson RM. Shared decision making for psychiatric medication management: a summary of its uptake, barriers and facilitators. *Int J Clin Pharm-Net.* 2021; 43: 759-763

56. Hoang K, Halpern-Felsher B, Brooks M, Blankenburg R. Shared Decision-making with Parents of Hospitalized Children: A Qualitative Analysis of Parents' and Providers' Perspectives. *Hospital Pediatrics.* 2020;10(11):977-985

57. Schoenfeld EM, Goff SL, Elia TR et al. Physician-identified barriers to and facilitators of shared decision-making in the Emergency Department: an exploratory analysis. *Emerg Med J*. 2019;36(6):346-354

58. van Til JA, Drossaert CH, Punter RA, Ijzerman MJ. The potential for shared decision-making and decision aids in rehabilitation medicine. *Journal of Rehabilitation Medicine*. 2010;42(6):598-604

59. Hamann J, Kohl S, McCabe R et al. What can patients do to facilitate shared decision making? A qualitative study of patients with depression or schizophrenia and psychiatrists. *Social Psychiatry & Psychiatric Epidemiology*. 2016;51(4):617-625

60. Tinetti ME, Naik AD, Dindo L et al. Association of Patient Priorities-Aligned Decision-Making with Patient Outcomes and Ambulatory Health Care Burden Among Older Adults with Multiple Chronic Conditions: A Nonrandomized Clinical Trial. *Jama Intern Med*. 2019;179(12):1688-1697

61. Miron-Shatz T, Golan O, Brezis M, Siegal G, Doniger GM. Shared decision-making in Israel: status, barriers, and recommendations. *Isr J Health Policy*. 2012;1(1):5-13

62. Glyn Elwyn Marie Anne VDW. A three-talk model for shared decision making: multistage consultation process. *BMJ*. 2017;359

63. Joseph-Williams N, Elwyn G, Edwards A. Knowledge is not power for patients: A systematic review and thematic synthesis of patient-reported barriers and facilitators to shared decision making. *Patient Education & Counseling*. 2014;94(3):291-309

64. Peek ME, Tang H, Cargill A, Chin MH. Are there racial differences in patients' shared decision-making preferences and behaviors among patients with diabetes? *Med Decis Making*. 2011;31(3):422-431

65. Durand MA, Carpenter L, Dolan H et al. Do Interventions Designed to Support Shared Decision-Making Reduce Health Inequalities? A Systematic Review and Meta-Analysis. *Plos One*. 2014;9(4): e94670

66. Abrines-Jaume N, Midgley N, Hopkins K et al. A qualitative analysis of implementing shared decision making in Child and Adolescent Mental Health Services in the United Kingdom: Stages and facilitators. *Clinical Child Psychology & Psychiatry.* 2016;21(1):19-31

67. Savelberg W, Boersma LJ, Smidt M et al. Does lack of deeper understanding of shared decision making explains the suboptimal performance on crucial parts of it? An example from breast cancer care. *Eur J Oncol Nurs*. 2019; 38: 92-97

68. Shepherd A, Shorthouse O, Gask L. Consultant psychiatrists' experiences of and attitudes towards shared decision making in antipsychotic prescribing, a qualitative study. *Bmc Psychiatry*. 2014; 14: 127-136

69. Jordan A, Joseph-Williams N, Edwards A, Holland-Hart D, Wood F. "I'd Like to Have More of a Say Because It's My Body": Adolescents' Perceptions Around Barriers and Facilitators to Shared Decision-Making. *J Adolescent Health*. 2019;65(5):633-642

70. Fullwood C, Kennedy A, Rogers A et al. Patients' experiences of shared decision making in primary care practices in the United Kingdom. Medical Decision Making an International *Journal of the Society for Medical Decision Making*. 2013;33(1):26-36

71. Haimowitz S, Ruiz-Cordell K, Joubin K, Sih-Meynier R, Schumacher U. A multi-level assessment of shared decision making: An evaluation of clinician and patient attitudes and behaviors and the identification of predictive profiles. *Cogent Medicine*. 2020;7(1):1-10

72. Munro S, Wilcox ES, Lambert LK et al. A survey of health care practitioners' attitudes toward shared decision-making for choice of next birth after cesarean. *Birth*. 2021; 00:1-15

73. Hamann J, Heres S. Adapting shared decision making for individuals with severe mental illness. P*sychiat Serv*. 2014;65(12):1483-1486

74. Liverpool S, Hayes D, Edbrooke-Childs J. An Affective-Appraisal Approach for Parental Shared Decision Making in Children and Young People's Mental Health Settings: A Qualitative Study. *Front Psychiatry*. 2021; 12:626848

75. Kehl KL, Landrum MB, Arora NK et al. Association of Actual and Preferred Decision Roles with Patient-Reported Quality of Care: Shared Decision Making in Cancer Care. *Jama Oncol*. 2015;1(1):50-58

76. Luo HW, Liu GH, Lu J, Xue D. Association of shared decision making with inpatient satisfaction: a cross-sectional study. *BMC Medical Informatics and Decision Making.* 2021;21(1):25-35

77. Frosch DL, May SG, Rendle KAS, Tietbohl C, Elwyn G. Authoritarian Physicians and Patients' Fear of Being Labeled 'Difficult' Among Key Obstacles to Shared Decision Making. *Health Affairs.* 2012;31(5):1030-1038

78. Boland L, McIsaac DI, Lawson ML. Barriers to and facilitators of implementing shared decision making and decision support in a paediatric hospital: A descriptive study. *Paed Child Healt-Can*. 2016;21(3): e17-21

79. Schapira MM, Faghri A, Jacobs EA et al. Communication and Shared Decision Making in the Breast Cancer Treatment Consultation: A Comparative Analysis of English- and Spanish-Speaking Patients. *MDM Policy & Practice*. 2019;4(2):1-10

80. Rosenberg-Yunger ZRS, Verweel L, Gionfriddo MR, MacCallum L, Dolovich L. Community pharmacists' perspectives on shared decision-making in diabetes management. *International Journal of Pharmacy Practice*. 2018;26(5):414-422

81. Roodbeen R, Vreke A, Boland G et al. Communication and shared decision-making with patients with limited health literacy; helpful strategies, barriers and suggestions for improvement reported by hospital-based palliative care providers. *Plos One.* 2020;15(6): e0234926

82. Clark BA, Virani A, Marshall SK, Saewyc EM. Conditions for shared decision making in the care of transgender youth in Canada. *Health Promot Int.* 2020;22(36):1-11

83. Hawley ST, Morris AM. Cultural challenges to engaging patients in shared decision making. *Patient Education & Counseling.* 2017;100(1):18-24

84. Nejati B, Lin C, Aaronson NK et al. Determinants of satisfactory patient communication and shared decision making in patients with multiple myeloma. *Psycho‐Oncology.* 2019;28(7):1490-1497

85. Henselmans I, van Laarhoven HWM, van Maarschalkerweerd P et al. Effect of a Skills Training for Oncologists and a Patient Communication Aid on Shared Decision Making About Palliative Systemic Treatment: A Randomized Clinical Trial. *The Oncologist.* 2020;25(3): e578-e588

86. Barton JL, Trupin L, Tonner C et al. English language proficiency, health literacy, and trust in physician are associated with shared decision making in rheumatoid arthritis. *The Journal of Rheumatology.* 2014;41(7):1290-1297

87. Maes-Carballo M, Martín-Díaz M, Mignini L et al. Evaluation of the Use of Shared Decision Making in Breast Cancer: International Survey. *International Journal of Environmental Research and Public Health*. 2021;18(4):2128-2142

88. Vaillancourt H, Legare F, Gagnon MP et al. Exploration of shared decision-making processes among dieticians and patients during a consultation for the nutritional treatment of dyslipidaemia. *Health Expect.* 2015;18(6):2764-2775

89. Bisset CN, Dames N, Oliphant R et al. Exploring shared surgical decision-making from the patient's perspective: is the personality of the surgeon important? *Colorectal Disease.* 2020;22(12):2214-2221

90. Wang M, Hung L, Lo Y. Glycemic control in type 2 diabetes: role of health literacy and shared decision-making. Patient Preference and Adherence. 2019; 13:871-879

91. Wigfall LT, Tanner AH. Health Literacy and Health-Care Engagement as Predictors of Shared Decision-Making Among Adult Information Seekers in the USA: a Secondary Data Analysis of the Health Information National Trends Survey. *J Canc Edu*. 2018;33(1):67-73

92. Schneider A, Körner T, Mehring M et al. Impact of age, health locus of control and psychological co-morbidity on patients' preferences for shared decision making in general practice. *Patient Educ Couns*. 2006;61(2):292-8

93. Holmes-Rovner M, Valade D, Orlowski C et al. Implementing shared decision-making in routine practice: barriers and opportunities. *Health Expect.* 2000;3(3):182-191

94. Koizumi T, Nara K, Hashimoto T et al. Influence of Negative Emotional Expressions on the Outcomes of Shared Decision Making During Oncofertility Consultations in Japan. *Journal of Adolescent & Young Adult Oncology*. 2018;7(4):504-508

95. Bot AGJ, Bossen JKJ, Herndon JH et al. Informed shared decision-making and patient satisfaction. *Psychosomatics*. 2014;55(6):586-594

96. Bradley E, Green D. Involved, inputting or informing: "Shared" decision making in adult mental health care. *Health Expect*. 2018;21(1):192-200

97. Stalnikowicz R, Brezis M. Meaningful shared decision-making: complex process demanding cognitive and emotional skills. *J Eval Clin Pract*. 2020;26(2):431-438

98. Hofstede SN, van Bodegom-Vos L, Wentink MM et al. Most important factors for the implementation of shared decision making in sciatica care: ranking among professionals and patients. *Plos One.* 2014;9(4): e94176

99. Sonntag U, Wiesner J, Fahrenkrog S et al. Motivational interviewing and shared decision making in primary care. *Patient Education & Counseling*. 2012;87(1):62-66

100. Ousseine YM, Durand M, Bouhnik A, Smith A», Mancini J. Multiple health literacy dimensions are associated with physicians' efforts to achieve shared decision-making. *Patient Educ Couns.* 2019;102(11):1949-1956

101. Berger-Hoger B, Liethmann K, Muhlhauser I, Haastert B, Steckelberg A. Nurse-led coaching of shared decision-making for women with ductal carcinoma in situ in breast care centers: A cluster randomized controlled trial. *Int J Nurs Stud.* 2019; 93:141-152

102. Tam-Seto L, Versnel J. Occupational Therapy Shared Decision Making in Adolescent Mental Health. *Occupational Therapy in Mental Health.* 2015;31(2):168-186

103. Butterworth JE, Campbell JL. Older patients and their GPs: shared decision making in enhancing trust. *British Journal of General Practice*. 2014;64(628): e709-718

104. Tariman JD, Mehmeti E, Spawn N et al. Oncology Nursing and Shared Decision Making for Cancer Treatment. *Clin J Oncol Nurs*. 2016;20(5):560-563

105. Evong Y, Chorney J, Ungar G, Hong P. Perceptions and observations of shared decision making during pediatric otolaryngology surgical consultations. *Journal of Otolaryngology - Head and Neck Surgery.* 2019;48(1):28

106. Davis K, Haisfield L, Dorfman C, Krist A, Taylor KL. Physicians' attitudes about shared decision making for prostate cancer screening. *Fam Med.* 2011;43(4):260-266

107. Young HN, Bell RA, Epstein RM, Feldman MD, Kravitz RL. Physicians' shared decision-making behaviors in depression care. *Archives of Internal Medicine.* 2008;168(13):1404-8

108. Fukui S, Salyers MP, Matthias MS et al. Predictors of shared decision making and level of agreement between consumers and providers in psychiatric care. *Community Mental Health Journal.* 2014;50(4):375-82

109. van den Brink-Muinen A, Spreeuwenberg P, Rijken M. Preferences and experiences of chronically ill and disabled patients regarding shared decision-making: does the type of care to be decided upon matter? *Patient Education & Counseling*. 2011;84(1):111-7

110. Spies CD, Schulz CM, Weiss-Gerlach E et al. Preferences for shared decision making in chronic pain patients compared with patients during a premedication visit. *Acta Anaesth Scand.* 2006;50(8):1019-26

111. Baig AM, Humayaun A, Mehmood S et al. Qualitative exploration of factors associated with shared decision-making in diabetes management: a health care provider's perspective. Int J Qual Health Care. 2020;32(7):464-469

112. Peek ME, Odoms-Young A, Quinn MT et al. Race and shared decision-making: perspectives of African-Americans with diabetes. *Soc Sci Med.* 2010;71(1):1-9

113. Hamann J, Buhner M, Rusch N. Self-Stigma and Consumer Participation in Shared Decision Making in Mental Health Services. *Psychiatric services: a journal of the American Psychiatric Association*. 2017;68(8):783-788

114. Barker C, Dunn S, Moore GP et al. Shared decision making during antenatal counselling for anticipated extremely preterm birth. *Paed Child Healt-Can.* 2019;24(4):240-249

115. Mahone IH, Farrell S, Hinton I et al. Shared decision making in mental health treatment: qualitative findings from stakeholder focus groups. *Arch Psychiat Nurs*. 2011;25(6):e27-36

116. Bouma AB, Tiedje K, Poplau S et al. Shared decision making in the safety net: where do we go from here? *The Journal of the American Board of Family Medicin*e. 2014;27(2):292-294

117. Moleman M, Regeer BJ, Schuitmaker-Warnaar TJ. Shared decision-making and the nuances of clinical work: Concepts, barriers and opportunities for a dynamic model. *J Eval Clin Pract.* 2020:1-9

118. Brogan P, Hasson F, McIlfatrick S. Shared decision-making at the end of life: A focus group study exploring the perceptions and experiences of multi-disciplinary healthcare professionals working in the home setting. *Palliative Med*. 2018;32(1):123-132

119. Vedasto O, Morris B, Furia FF. Shared decision-making between health care providers and patients at a tertiary hospital diabetic Clinic in Tanzania. *Bmc Health Serv Res.* 2021;21(1):8-15

120. Suurmond J, Seeleman C. Shared decision-making in an intercultural context. Barriers in the interaction between physicians and immigrant patients. *Patient Education & Counseling.* 2006;60(2):253-9

121. Mariani E, Vernooij-Dassen M, Koopmans R, Engels Y, Chattat R. Shared decision-making in dementia care planning: barriers and facilitators in two European countries. A*ging Ment Health*. 2017;21(1):31-39

122. Acerini CL, Segal D, Criseno S et al. Shared Decision-Making in Growth Hormone Therapy-Implications for Patient Care. *Front Endocrinol.* 2018; 9:688-702

123. Peek ME, Drum M, Cooper LA. The Association of Patient Chronic Disease Burden and Self-Management Requirements with Shared Decision Making in Primary Care Visits. *Health Services Research & Managerial Epidemiology*. 2014; 1:1-8

124. Kraaij GE, Vermeulen FM, Smeets PMG, Smets EMA, Spuls PI. The current extent of and need for shared decision making in atopic dermatitis and psoriasis in the Netherlands: an online survey study amongst patients and physicians. *J Eur Acad Dermatol.* 2020;34(11):2574-2583

125. Berger S, Braehler E, Ernst J. The health professional-patient-relationship in conventional versus complementary and alternative medicine. A qualitative study comparing the perceived use of medical shared decision-making between two different approaches of medicine. *Patient Education & Counseling*. 2012;88(1):129-37

126. Shen H, Lin C, Hoffmann T et al. The relationship between health literacy and perceived shared decision making in patients with breast cancer. *Patient Educ Couns.* 2019;102(2):360-366

127. Smith SG, Pandit A, Rush SR, Wolf MS, Simon CJ. The Role of Patient Activation in Preferences for Shared Decision Making: Results from a National Survey of U.S. Adults. *J Health Commun.* 2016;21(1):67-75

128. Pellerin M, Elwyn G, Rousseau M et al. Toward shared decision making: using the OPTION scale to analyze resident-patient consultations in family medicine. *Acad Med*. 2011; 86(8): 1010-1018

129. Keij SM, van Duijn-Bakker N, Stiggelbout AM, Pieterse AH. What makes a patient ready for Shared Decision Making? A qualitative study. *Patient Educ Couns.* 2021;104(3):571-577

130. Luciano M, Sampogna G, Del Vecchio V et al. When does shared decision making is adopted in psychiatric clinical practice? Results from a European multicentric study. *European Archives of Psychiatry and Clinical Neuroscience*. 2020;270(6):645-653

131. Astbury R, Shepherd A, Cheyne H. Working in partnership: the application of shared decision-making to health visitor practice. *J Clin Nurs.* 2017;26(1-2):215-224
